# Efficient Cleavage of pUC19 DNA by Tetraaminonaphthols

**DOI:** 10.1002/open.202400157

**Published:** 2024-10-25

**Authors:** Catharina Kost, Ute Scheffer, Elisabeth Kalden, Michael Wilhelm Göbel

**Affiliations:** ^1^ Institut für Organische Chemie und Chemische Biologie Goethe-Universität, Frankfurt am Main Max-von-Laue-Str. 7 D-60438 Frankfurt am Main Germany

**Keywords:** Anion receptor, Azo naphthol, DNA cleavage, Enzyme mimic, Organocatalysis

## Abstract

In an attempt to create models of phosphodiesterases, we previously investigated bis(guanidinium) naphthols. Such metal‐free anion receptors cleaved aryl phosphates and also plasmid DNA. Observed reaction rates, however, could not compete with those of highly reactive metal complexes. In the present study, we have replaced the guanidines by ethylene diamine side chains which accelerates the plasmid cleavage by compound **13** significantly (1 mM **13**: t1/2=22 h). Further gains in reactivity are achieved by azo coupling of the naphthol unit. The electron accepting azo group decreases the p*K*_a_ of the hydroxy group. It can also serve as a dye label and a handle for attaching DNA binding moieties. The resulting azo naphthol **17** not only nicks (1 mM **17**: t1/2~1 h) but also linearizes pUC19 DNA. Although the high reactivity of **17** seems to result in part from aggregation, in the presence of EDTA azo naphthol **17** obeys first order kinetics (1 mM **17**: t1/2=4.8 h), reacts four times faster than naphthol **13** and surpasses by far the former bis(guanidinium) naphthols **4** and **5**.

## Graphical abstract

Anion receptor **4** was shown previously to cleave phosphodiesters by nucleophilic substitution. It also nicks plasmid DNA to some extent. We have now replaced the guanidines by ethylene diamine side chains. By this modification, the resulting tetraamine **13** becomes an effective DNA cleaver (R=H). Azo coupling of **13** further boosts the reactivity of the resulting naphthols **16** and **17** (**16**: R=4‐carboxyphenylazo; **17**: R=4‐methoxyphenylazo).

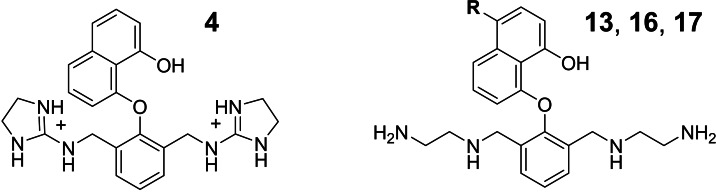

Nature has chosen phosphodiester groups as linker units of DNA on the basis of exceptional kinetic stability.^[1]^ Deprotonated at physiologic pH, these groups are protected from nucleophilic attack by their negative charge. Accordingly, the half‐life of non‐activated phosphodiesters in water at 25 °C is in the range of millions of years.^[2]^ Enzymes, on the other hand, are still able to cleave them with surprising ease, attaining rate increases of 17 orders of magnitude and more.^[2]^ Over the last decades, much has been learned on enzymes with respect to structures and modes of action. However, the de‐novo construction of equally reactive enzyme mimics has remained elusive.

The majority of phosphodiesterases contain two or more metal ions in their active site.[^3^, ^4^, ^5^, ^6^] These ions mainly act as Lewis acids and reduce the negative charge density of phosphates. In addition, they help to activate the attacking nucleophiles and stabilize leaving groups.[^7^, ^8^, ^9^] Most enzyme models, therefore, rely on metal complexes.[^10^, ^11^, ^12^, ^13^] The most effective artificial system to cleave DNA is based on highly charged Ce^4+^ ions.[^14^, ^15^, ^16^, ^17^, ^18^, ^19^] In contrast, human topoisomerase I and also several tyrosine recombinases do not depend on metal ions.^[20]^ They reversibly cleave and ligate supercoiled DNA using tyrosine hydroxy groups as nucleophiles. Two cationic arginine side chains replace Mg^2+^ ions in their role as phosphate activating electrophiles and a histidine is assumed to act as general acid for leaving group protonation.^[21]^ A comparable role as activating electrophiles has been assigned to Arg–35 and Arg–87 in staphylococcal nuclease.^[22]^

When we launched a program on artificial nucleases years ago, we abstained from metal ions. Instead, we arranged functional groups like amines, guanidines and alcohols in such a way that increasing reactivity against phosphodiesters should emerge.[^23^, ^24^] Inspired by the twin‐arginine motiv of topoisomerase I and staphylococcal nuclease, we started to examine the guanidinium alcohol **1**.^[25]^ In aprotic solvents, this compound formed ion pair complexes with catechol cyclic phosphate **2** (Figure 1). Compared to uncharged alcohols, the phosphorylation of **1** was million‐fold accelerated.^[25]^ However, compound **1** already failed to cleave BNPP **3** (bis(4‐nitrophenyl)‐phosphate), a still highly activated substrate but less reactive than phosphate **2**. We tried next to broaden the scope of possible substrates by optimizing the side chain structure. In contrast to compound **1**, the guanidinium naphthols **4** and **5** reacted selectively by *O*‐phosphorylation (e. g. forming product **6** from BNPP) with activated phosphodiesters in DMF and in DMF water mixtures.^[26]^ Compound **5** likewise cleaved plasmid DNA. When pUC19 was incubated with 5 mM of **5**, 32 % of the supercoiled plasmid was nicked at pH 7.0 and 49 % at pH 8.5 (37 °C, 20 h). Double strand breakage did not occur. In a third study, compound **5** was attached to a minor groove binding hairpin polyamide.^[27]^ While this modification enhanced DNA affinities by three orders of magnitude, again not more than 50 % of the plasmid was nicked within 20 h by guanidinium naphthol **7**. Thus, although compounds **5** and **7** are active, they fall behind in comparison with other DNA cleaving DNAzymes[^28^, ^29^] or metal complexes.[^30^, ^31^, ^32^, ^33^, ^34^, ^35^] A clear weakness of guanidinum alcohols **1**–**7** becomes visible by inspection of molecular models. The two cations are properly placed to accommodate tetraedric oxoanions. However, when the naphthol OH attacks phosphodiesters by an in‐line mechanism, the leaving group is too far away from the guanidinium ions to enable proton transfer. This fact is less critical when good leaving groups like 4‐nitrophenolate are involved but becomes crucial when DNA has to be cleaved. In the present study, we therefore replaced the guanidines by ethylene diamine or by TREN (tris(2‐aminoethyl)amine). The flexible side chains of the resulting compounds **13** and **15** can wrap around the substrate to find a favorable position for leaving group protonation (Scheme 1). As shown below, this change has major consequences for kinetics.

**Figure 1:**
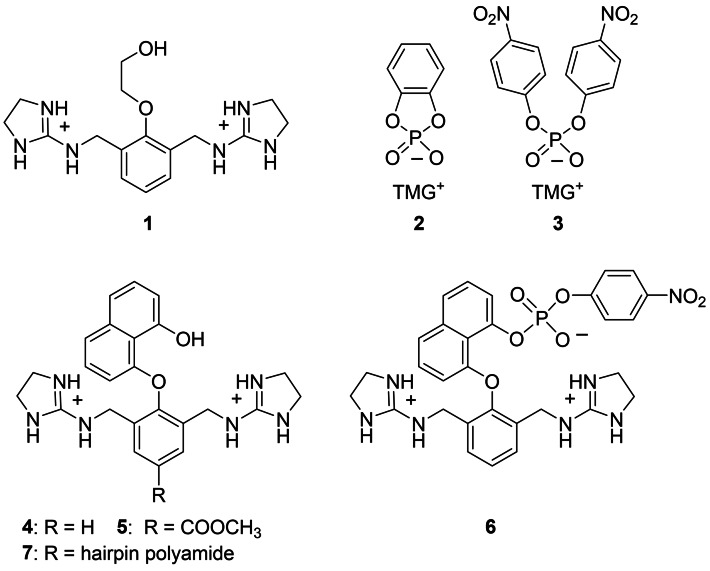
Bisguanidinium alcohols **1**–**7** investigated previously. Counterion: picrate or chloride; TMG: 1,1,3,3‐tetramethylguanidinium. For structural details of compound **7** see ref. [27].

**Scheme 1:**
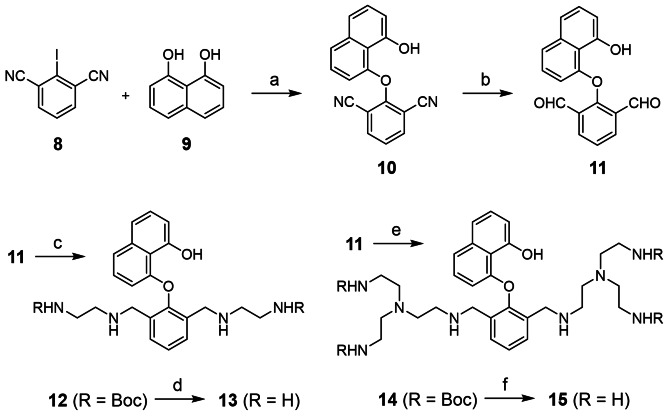
Synthesis of the polyamino naphthols **13** and **15**. a) NaH, DMF, 90 °C, see ref. [26]. b) DIBAL−H, CH_2_Cl_2_, −78 °C→0 °C, 46 %. c) Boc‐protected ethylenediamine **S1**, NaBH_4_, MeOH, room temp., 91 %. d) Trifluoroacetic acid, CH_2_Cl_2_, room temp., then anion exchange resin, 71 % as hydrochloride salt. e) Boc‐protected TREN **S2**, NaBH_4_, MeOH, 55 °C, 35 %. f) See condition d, 75 % as hydrochloride salt.

The synthesis of **13** and **15** starts from compounds **8** and **9** by nucleophilic aromatic displacement as published previously.^[26]^ The resulting dinitrile **10** (40–62 %) is then converted with DIBAL−H into dialdehyde **11** (46 %). Reductive amination with Boc‐protected ethylene diamine or TREN leads to intermediates **12** (91 %) and **14** (35 %) which can be purified by column chromatography. Acid treatment deprotects the amino groups and ion exchange finally converts compounds **13** (71 %) and **15** (75 %) into water soluble hydrochloride salts. For the OH group of **13** a p*K*_a_ value of 11.1±0.1 was determined (UV/VIS titration, Figure S1).

Figure 2 shows the cleavage of pUC19 plasmids by compound **13**. At high concentrations of **13**, almost complete conversion into the nicked form occurs within 18 h. In the presence of 1 mM of **13**, the reaction obeys a first‐order rate law (Figures 3 and Figure S11) with *k*_obs_=5.2±0.3×10^−4^ min^−1^, corresponding to a half‐life of supercoiled DNA of 22 h. Compared to previous results with guanidinium naphthol **5**, this constitutes a significant improvement. Cleavage is almost independent from pH in the range from 6.5–9 (Figure S3). Analogous experiments with compound **15** showed similar but not superior effects. In general, it was more complicated to synthesize and to handle this highly charged compound. Plasmid cleavage in the presence of **15** also suffered from blurred bands and precipitation effects. Therefore, we focused our studies on **13** and related compounds. To rule out metal ion contaminations of **13**, we added EDTA to the cleavage experiments. Even at high concentrations of 10 mM no retardation is observed (Figure S4). Addition of Mg^2+^ does not affect the reaction whereas Zn^2+^ (0.05–2 mM) retards plasmid cleavage (Figure S5).

**Figure 2:**
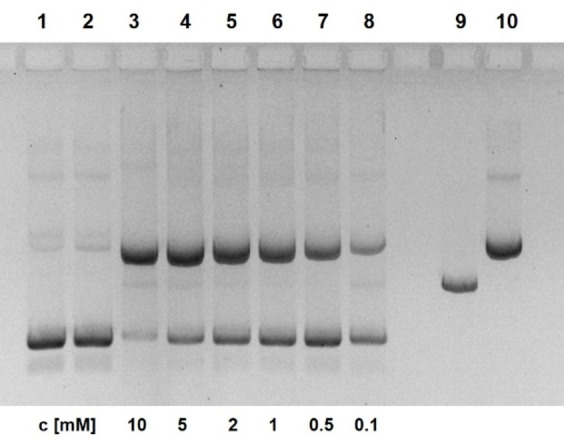
Cleavage of pUC19 plasmid DNA by compound **13** at pH 7.0. Lane 1 (from left): control 0 h; lane 2: control 20 h; lane 3: 10 mM **13**; lane 4: 5 mM **13**; lane 5: 2 mM **13**; lane 6: 1 mM **13**; lane 7: 0.5 mM **13**; lane 8: 0.1 mM **13**; lane 9: linearized plasmid; lane 10: nicked plasmid (22.5 nM DNA, 50 mM HEPES NaOH, 37 °C, 18 h; electrophoresis on 1 % agarose gel, ethidium staining; inverted gray scale. For details see Supporting Information).

**Figure 3:**
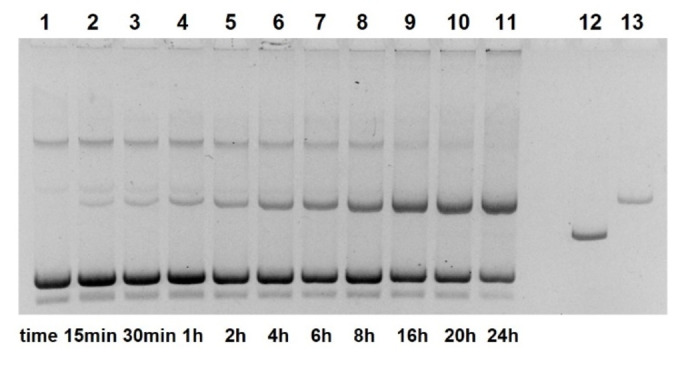
Cleavage of pUC19 DNA by compound **13** (1 mM, pH 7.0) as a function of time. Lane 1 (from left): control 0 h; lane 2: 15 min; lane 3: 30 min; lane 4: 1 h; lane 5: 2 h; lane 6: 4 h; lane 7: 6 h; lane 8: 8 h; lane 9: 16 h, lane 10: 20 h; lane 11: 24 h; lane 12: linearized plasmid; lane 13: nicked plasmid (22.5 nM DNA, 50 mM HEPES NaOH, 37 °C, electrophoresis on 1 % agarose gel, ethidium staining; inverted gray scale. See Supporting Information).

Due to the naphthol structure, compound **13** is susceptible to azo coupling (Figure 4). This reaction can introduce a handle for attaching DNA binding moieties. The azo group also constitutes a pH‐dependent dye label and lowers the p*K*_a_ of the hydroxy group. As expected, a basic solution (2 M KOH in MeOH) of **12** reacts with the diazonium ion from 4‐amino benzoic acid forming an azo naphthol in 46 % yield after chromatography. Removal of Boc then leads to compound **16** (84 %). The red color of azo naphthol **16** in neutral solution changes to magenta under basic conditions (Figure S2). By UV/VIS spectrometric titration, a p*K*_a_ value of 8.9±0.1 was determined (Figure S1). The ^13^C NMR of **16**, however, shows a signal in the carbonyl region (187 ppm) which is not compatible with the structure shown in Figure 4. 2‐ and 4‐azo naphthols are known to be in equilibrium with a second tautomeric form, the hydrazone of the related ortho‐ or para‐naphthoquinones (Figure 4), lacking the nucleophilic OH group.^[36]^ Electron acceptors in the second aryl ring shift the equilibrium in favor of this tautomer.[^37^, ^38^] Since the hydroxy group is crucial for the intended mechanism of DNA cleavage, we replaced the carboxy group in **16** by an electron donating methoxy residue. The resulting compound **17** is obtained by azo coupling of 4‐methoxyphenyl diazonium chloride with naphthol **12** (36 %) and removal of Boc (77 %). According to ^13^C NMR data, the azo naphthol form is the preferred tautomer of **17** because no signals beyond 159 ppm are visible. Unfortunately, UV/VIS spectrometric titration of **17** did not provide consistent p*K*_a_ data.

**Figure 4:**
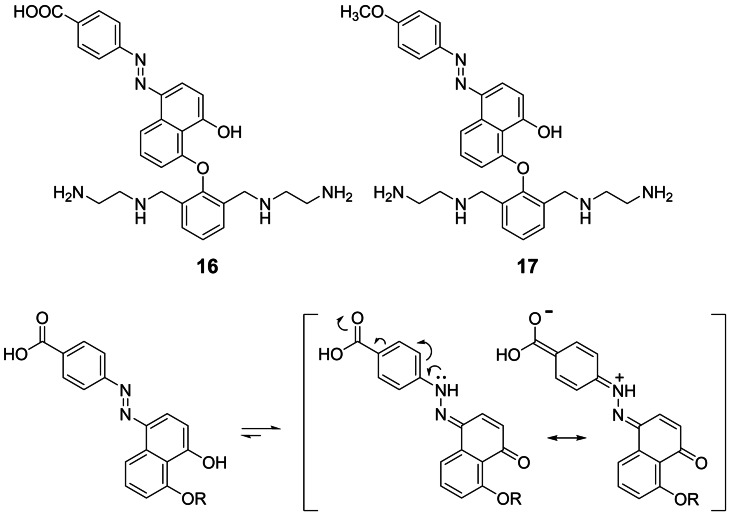
Top: Structures of the azo modified polyamino naphthols **16** and **17**. Bottom: Possible tautomeric forms of compound **16** and impact of electron withdrawing residues on the azo/hydrazone‐equilibrium.

Compared to compound **13**, the azo moiety of naphthol **16** accelerates the cleavage of pUC19, in spite of the unfavorable tautomer distribution. At higher concentrations, significant amounts of linear DNA are formed and 0.5 mM of **16** is sufficient to nick more than 80 % of the plasmid within 20 h (Figure 5, lane 7). The formation of linear DNA implies that breakage of the second strand must have occurred within 15 base pairs from the first cut.^[39]^ The time course of the reaction was determined at a cleaver concentration of 1 mM (Figures 6 and Figure S12). The half‐life of supercoiled plasmid under such conditions is in the range of 3 h. Even at this low concentration of **16**, some linear DNA is formed within 20 h. It should be noted, however, that the reaction does not obey a first order rate law (Figure S12) and addition of EDTA significantly retards DNA cleavage (see below). In contrast to **13**, the amount of nicked plasmid decreases with pH values changing from 6.5–9 (Figure S6).

**Figure 5:**
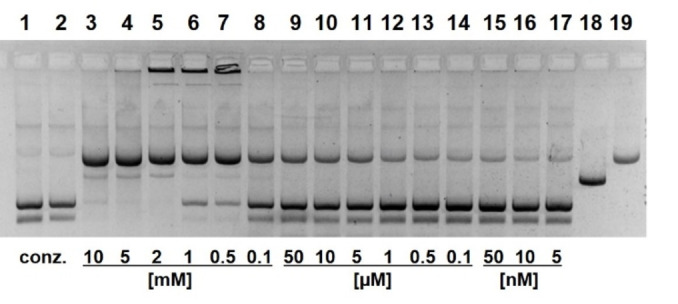
Cleavage of pUC19 plasmid DNA by compound **16** at pH 7.0. Lane 1: control 0 h; lane 2: control 20 h; lane 3: 10 mM **16**; lane 4: 5 mM **16**; lane 5: 2 mM **16**; lane 6: 1 mM **16**; lane 7: 0.5 mM **16**; lane 8: 0.1 mM **16**; lane 9: 50 μM **16**; lane 10: 10 μM **16**; lane 11: 5 μM **16**; lane 12: 1 μM **16**; lane 13: 0.5 μM **16**; lane 14: 0.1 μM **16**;. lane 15: 0.05 μM **16**; lane 16: 0.01 μM **16**; lane 17: 0.005 μM **16**; lane 18: linearized plasmid; lane 19: nicked plasmid. (22.5 nM DNA, 50 mM HEPES NaOH, 37 °C, 20 h; electrophoresis on 1 % agarose gel, ethidium staining; inverted gray scale. See Supporting Information).

**Figure 6:**
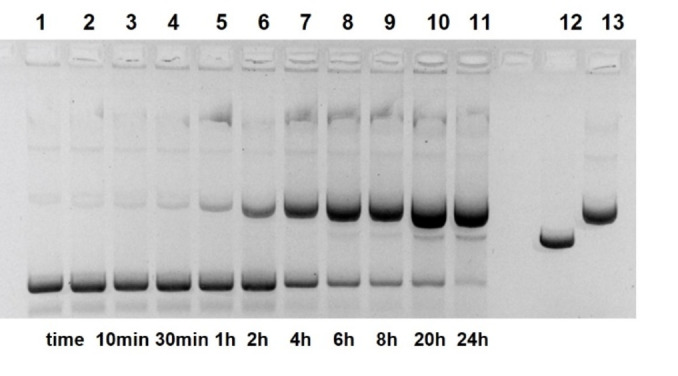
Cleavage of pUC19 plasmid DNA by compound **16** as a function of time. (1 mM of **16**, pH 7.0, 37 °C). Lane 1: control 0 h; lane 2: control 20 h; lane 3: 10 min; lane 4: 30 min; lane 5: 1 h; lane 6: 2 h; lane 7: 4 h; lane 8: 6 h; lane 9: 8 h; lane 10: 20 h; lane 11: 24 h; lane 12: linearized plasmid; lane 13: nicked plasmid (22.5 nM DNA, 50 mM HEPES NaOH; electrophoresis on 1 % agarose gel, ethidium staining; inverted gray scale. See Supporting Information).

Due to the higher fraction of the azo naphthol tautomer, compound **17** should react with DNA even faster (Figure 7). Indeed, pUC19 cleavage by **17** in concentrations from 0.5–10 mM not only yields much linear DNA. In addition, the total band intensity of cleavage products declines. This behavior normally results from unspecific fragmentation of the linear DNA.^[40]^ Such products will have a broad distribution of lengths and thus do not show defined bands in the gel. Significant cleavage is observed at concentrations of **17** down to 1 μM and below (Figure 7, lanes 12–14). A major attenuation reproducibly results when **17** is diluted from 0.5–0.1 mM. We think this is caused by hydrophobic aggregation of **17** at higher concentrations, a well documented phenomenon of amphiphilic azo dyes in water.[^41^, ^42^, ^43^] Multimeric aggregates of **17** are expected to show increased reactivity. Cleavage kinetics induced by 1 mM of **17** is shown in Figure 8. Plasmid nicking is very fast (~50 % conversion in 1 h) and linear DNA is already detectable after 6 h. The initial phase of the reaction has been checked by special kinetic runs: Approximately 30 % of pUC19 is nicked within the first 30 min (1 mM **17**, pH 7.0; Figures S7 and S14). However, the reaction does not follow first‐order kinetics and slows down with time (Figure S13). When the stock solution of **17** (20 mM) is diluted to 1 mM, a time‐dependent dissociation of aggregates can be expected which may explain the negative deviation from first‐order kinetics.

**Figure 7:**
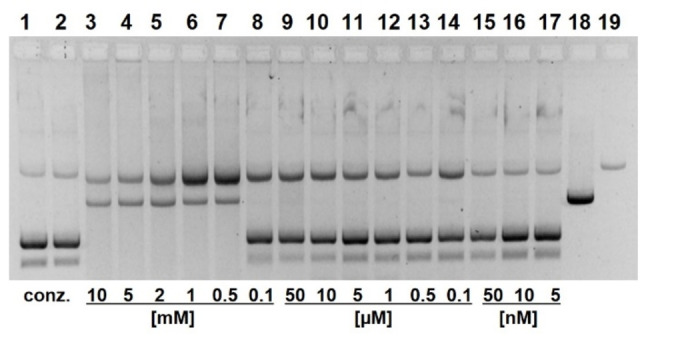
Cleavage of pUC19 plasmid DNA by compound **17** at pH 7.0. Lane 1: control 0 h; lane 2: control 20 h; lane 3: 10 mM **17**; lane 4: 5 mM **17**; lane 5: 2 mM **17**; lane 6: 1 mM **17**; lane 7: 0.5 mM **17**; lane 8: 0.1 mM **17**; lane 9: 50 μM **17**; lane 10: 10 μM **17**; lane 11: 5 μM **17**. lane 12: 1 μM **17**; lane 13: 0.5 μM **17**; lane 14: 0.1 μM **17**; lane 15: 0.05 μM **17**; lane 16: 0.01 μM **17**; lane 17: 0.005 μM **17**; lane 18: linearized plasmid; lane 19: nicked plasmid. (22.5 nM DNA, 50 mM HEPES NaOH, 37 °C, 20 h; electrophoresis on 1 % agarose gel, ethidium staining; inverted gray scale. See Supporting Information).

**Figure 8:**
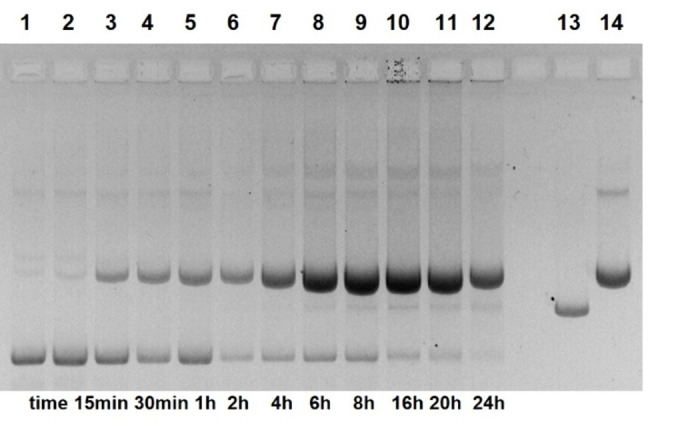
Cleavage of pUC19 plasmid by azo naphthol **17** as a function of time. (1 mM of **17**, pH 7.0, 37 °C). Lane 1: control 0 h; lane 2: control 20 h; lane 3: 15 min; lane 4: 30 min; lane 5: 1 h; lane 6: 2 h; lane 7: 4 h; lane 8: 6 h; lane 9: 8 h; lane 10: 16 h; lane 11: 20 h; lane 12: 24 h; lane 13: linearized plasmid; lane 14: nicked plasmid (22.5 nM DNA, 50 mM HEPES NaOH; electrophoresis on 1 % agarose gel, ethidium staining; inverted gray scale. See Supporting Information).

Although we had no evidence from X‐ray fluorescence data for metal ion contaminations, we treated the aqueous solutions of **16** and **17** with Chelex beads. Unfortunately, both azo naphthols within short time are quantitatively absorbed by the resin. Even small amounts of EDTA (20–100 μM) cause a significant retardation of DNA cleavage with both azo naphthols **16** and **17**, in contrast to compound **13** (Figure S8). Higher concentrations (0.5 mM) slow down the reaction even more (Figure S9). The complete absorption on Chelex resin points to a strong affinity to iminodiacetic acid and EDTA. To guarantee the absence of critical metal ions, we repeated the cleavage kinetics with **17** (1 mM) in the presence of 100 μM EDTA (Figure 9). Strikingly, the reaction now obeys first‐order kinetics and does no longer show the retardation over time (Figure S15). As a possible explanation we assume that in presence of EDTA aggregates of **17** rapidly dissolve. The half‐life of supercoiled plasmid under such conditions rises from 1 h to 4.8 h (*k*_obs_=2.4±0.3×10^−3^ min^−1^) but small amounts of linear DNA are still formed. As in the case of **13**, addition of Mg^2+^ has no impact whereas Zn^2+^ retards the cleavage of pUC19 by compound **17** (Figure S10).

**Figure 9:**
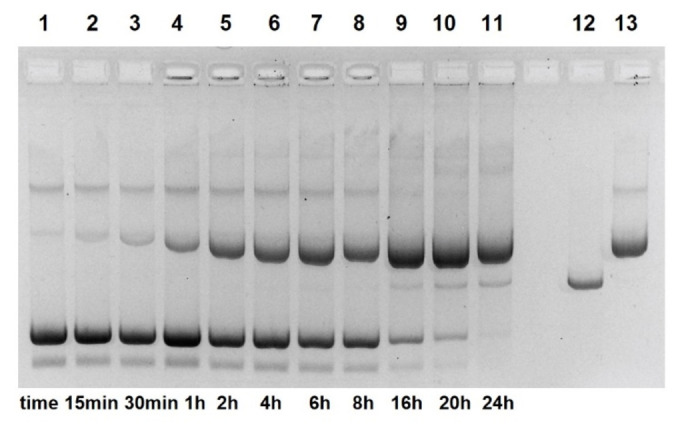
Influence of EDTA on pUC19 cleavage kinetics by azo naphthol **17**. (100 μM EDTA, 1 mM of **17**, pH 7.0, 37 °C). Lane 1: control 0 h; lane 2: 15 min; lane 3: 30 min; lane 4: 1 h; lane 5: 2 h; lane 6: 4 h; lane 7: 6 h; lane 8: 8 h; lane 9: 16 h; lane 10: 20 h; lane 11: 24 h; lane 12: linearized plasmid; lane 13: nicked plasmid (22.5 nM DNA, 50 mM HEPES NaOH; electrophoresis on 1 % agarose gel, ethidium staining; inverted gray scale. See Supporting Information)

## Conclusions

The aim of this study was to find a rationale for augmenting the DNA cleaving reactivity of bisguanidinium naphthols **4** and **5**. In a first step replacement of guanidines by ethylene diamine units led to compound **13**. In contrast to **4** and **5**, naphthol **13** (10 mM) fully converts supercoiled DNA into the nicked form. By conversion of **13** into azo naphthol **16** the reactivity rises further and plasmid half‐life drops from 22 h (1 mM **13**) to 3 h (1 mM **16**). Compound **16** still has the drawback of an unfavorable tautomer ratio, a problem no longer present in azo naphthol **17**. This compound by far surpasses our previous DNA cleaving anion receptors. It nicks 50 % of supercoiled plasmid within 60 min (1 mM **17**) and at 10 μM concentration (Figure 7) it gives comparable effects as 10 mM of guanidinium naphthol **5**. Site‐specific cleavage of single stranded DNA, on the other hand, has not been achieved with compounds **13**, **16**, and **17**. Such reactions are the domain of DNAzymes[^28^, ^29^] and of Ce^4+^‐based systems such as ARCUT.[^16^, ^17^, ^18^] In contrast, the large majority of DNA cleavage studies uses plasmids. pUC19, for example, consists of 5372 nucleotides in two strands. Cleavage just of a single internucleotidic bond by hydrolysis or by other mechanisms converts supercoiled into open circle DNA. The formation of product bands, therefore, results from sampling over thousands of similar reaction pathways. This statistical factor combined with superhelical strain makes plasmid DNA a rather sensitive substrate. Working with dinuclear Ce^4+^complexes (25 μM), Que observed nicking of plasmids with half‐lives less than 80 min.^[14]^ The cerium catalyst also induced double strand breaks and cleaved restriction fragments. More recently, Scrimin investigated plasmid cleavage by peptide decorated gold nanoparticles. A specific peptide containing arginine, serine and a TACN residue was already active in metal‐free form. The corresponding Zn^2+^ complex nicked the pBR322 plasmid with t1/2 around 36 min.^[34]^ Cooperative effects of metal complexes and organic cations are also known from other studies.[^44^, ^45^, ^46^] A number of metal‐free plasmid cleavers has also appeared in literature[^47^, ^48^, ^49^, ^50^, ^51^, ^52^, ^53^, ^54^, ^55^, ^56^, ^57^, ^58^, ^59^, ^60^, ^61^, ^62^, ^63^]: Lu and Xu, for example, presented a simple but remarkably effective compound consisting of a central TACN ring equipped with hydroxy and guanidinium side chains.^[47]^ It showed saturation kinetics with *k*_max_=2.7×10^−3^ min^−1^ (t1/2=4.3 h). Even faster cleavage was observed when the DNA affinity of related compounds was increased by an anthraquinone residue (t1/2=1.5 h).^[48]^ The highest nicking rates of metal‐free DNA cleavers was reported for a cyclic peptide built from two arginines, a serine and an anthraquinone residue (*k*_obs_=2.7×10^−2^ min^−1^, t1/2=26 min).^[49]^ Several guanidinium based catalysts have been presented by Salvio. Although not tested for plasmid DNA, they are successful cleavers of phosphodiester model substrates.[^50^, ^51^] With a plasmid half‐life around 1 h (1 mM **17**; faster with 10 mM), azo naphthol **17** compares favorably with other DNA cleaving compounds. However, the deviation from first‐order kinetics indicates that oligomeric aggregates may still contribute to the high reactivity. The retarding effect of EDTA presumably can be attributed to a fast dissociation of aggregates and not to a capture of catalytically active metal ions. A final proof of this idea, however, must await further studies. In the presence of EDTA, azo naphthol **17** safely acts as a metal‐free agent. With a substrate half‐life of 4.8 h (1 mM **17**, 0.1 mM EDTA) it is still a respectable DNA cleaver.

## Conflict of Interests

The authors declare no conflict of interest.

## Supporting information

As a service to our authors and readers, this journal provides supporting information supplied by the authors. Such materials are peer reviewed and may be re‐organized for online delivery, but are not copy‐edited or typeset. Technical support issues arising from supporting information (other than missing files) should be addressed to the authors.

Supporting Information

## Data Availability

The data that support the findings of this study are available in the supplementary material of this article.
