# Supplementary material for: Efficient Cleavage of pUC19 DNA by Tetraaminonaphthols
Source: ChemistryOpen. 2024 Oct 25;14(2):e202400157. doi: 10.1002/open.202400157 (PMC11808266; doi:10.1002/open.202400157)
Supplement: Supplementary file 1 — Supporting Information [file OPEN-14-e202400157-s001.pdf]

ChemistryOpen

Supporting Information

Efficient Cleavage of pUC19 DNA by Tetraaminonaphthols

Catharina Kost, Ute Scheffer, Elisabeth Kalden, and Michael Wilhelm Göbel*

Efficient Cleavage of pUC19 DNA by Tetraaminonaphthols

Catharina Kost, Ute Scheffer, Elisabeth Kalden, and Michael W. Göbel*

Supporting Information

Table of contents:

General....S2

Synthesis of Polyaminonaphthols....S2

Determination of pK_a Values....S13

DNA Cleavage Assay....S14

XRF Measurements....S21

NMR Spectra....S22

References....S36

General

All chemicals were reagent grade and used as purchased. Oxygen and water sensitive reactions were conducted under argon. Column chromatography: silica gel, 60 Å pore size, 0.04-0.063 mm particle size (Macherey-Nagel). Proton nuclear magnetic resonance (^{1}H NMR) and carbon nuclear magnetic resonance spectra (^{13}C-NMR) were recorded with a Bruker AV 400 (^{1}H: 400 MHz; ^{13}C: 101 MHz), Bruker AV 500 (^{1}H: 500 MHz; ^{13}C: 126 MHz) or Bruker DRX 600 (^{1}H: 600 MHz) spectrometer. Chemical shifts for protons are reported in parts per million (δ scale) and internally referenced to the proton resonances of the solvent (d_6-DMSO: δ 2.50, D_2O: δ 4.79). Chemical shifts for carbon are reported in parts per million (δ scale) and referenced to the carbon resonances of the solvent (d_6-DMSO: δ 39.52). Data are represented as follows: chemical shift, multiplicity (s = singlet, bs = broad singlet, d = doublet, t = triplet, q = quartet, m = multiplet, dd = double doublet), coupling constants in Hz, and integration. The IR spectra were recorded with a UATR Two FT-IR (Perkin Elmer). Mass spectra. ESI-MS spectra were obtained on a Fisons VG Plattform II, LC-ESI spectra on a Surveyor MSQ instrument (ThermoFisher). HRMS spectra were recorded on a MALDI LTQ Orbitrap mass spectrometer (ThermoFisher). The HRMS spectra of **S4** was recorded on an ESI-MS impact II (Bruker). The pK_a values were determined via UV-Vis titration on a NanoDrop 2000c spectrometer (ThermoFisher).

Synthesis of Polyaminonaphthols

2-Iodoisophthalonitrile 8

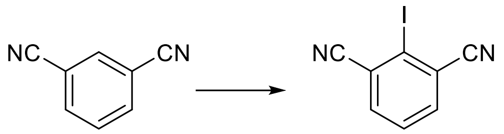

8

Isophthalonitrile (5.00 g, 39 mmol, 1 eq.) was dissolved in 350 mL of dry THF and cooled to -78 °C. LDA-solution (2M in THF, 21.5 mL, 43 mmol, 1.1 eq.) was added by use of a dropping funnel within 10 min. The resulting dark orange solution was stirred for 30 min. Then I_2 (9.90 g, 39 mmol, 1 eq.) was dissolved in dry THF (50 mL) and slowly added to the mixture. The reaction was stirred for 1 h and then warmed to RT. The solvent was evaporated, and the crude mixture was mixed with EtOAc and washed with saturated $Na_2S_2O_3$. The organic phase was dried over Na_2SO_4, and the solvent was evaporated. The crude product was purified by column chromatography (EtOAc/ cHex 1:1) and recrystallized (EtOAc/ cHex) to yield **8** in form of colorless needles (8.15 g, 82 %).

^{1}H-NMR (400 MHz, DMSO-d_6): δ[ppm] = 8.11 (d, J = 7.9 Hz, 2H, Ar-H), 7.77 (t, J = 7.9 Hz, 1H, Ar-H).

^{13}C-NMR (101 MHz, DMSO-d_6): δ[ppm] = 138.23, 129.71, 121.53, 118.81, 106.80.

IR (cm^{-1}): 3178.32 (w), 2233.43 (m), 1612.38 (m), 1403.86 (s), 1237.10 (m), 1027.00 (m), 802.53 (s), 704.33 (s), 516.83 (m).

MP: 208-209°C Ref.: 208-209°C. [2]

R_f = 0.59 (cHex/ EtOAc 1:1).

1,8-Dihydroxynaphthalene 9

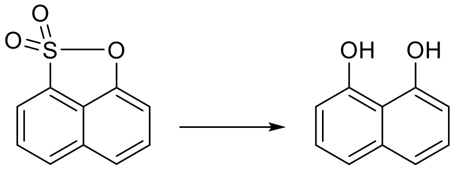

9

KOH (51.00 g, 0.91 mol, 15 eq.) was melted at 220-240 °C. 1-Naphthol-8-sulfonic acid sultone (12.5 g, 0.06 mol, 1 eq.) was carefully added under argon in small portions. The resulting dark green suspension was stirred for 1h. After cooling down, 150 mL of 6 M HCl was added to the mixture which was extracted with EtOAc. The combined organic phases were dried over Na_2SO_4 and the solvent was evaporated. The crude product was purified by column chromatography (cHex/ EtOAc 2:1) to yield **9** as a pale-yellow solid (4.89 g, 51 %).

^{1}H-NMR (400 MHz, DMSO-*d*$_6$): δ[ppm] = 10.83 (s, 2H, 2x OH), 7.27 -7.25 (m, 4H, Ar-H), 6,71 (m, 2H, Ar-H).

^{13}C-NMR (101 MHz, DMSO-*d*$_6$): δ[ppm] = 154.01, 136.54, 126.84, 118.82, 114.47, 108.37.

IR (cm^{-1}): 3127.92 (m), 1611.28 (s), 1402.57, 1276.87 (s), 1024.28 (s), 811.15 (s), 745.13 (s), 593.44 (m).

MS (ESI): m/z calcd. for $C_{10}H_7O_2$([M-H$^+$]): 159.05; found: 159.24.

HRMS: m/z calcd. for $C_{10}H_8O_2$ ([M$^{\bullet+}$]): 160.05188; found: 160.05185.

MP: 142-143°C Ref.: 142-143°C.[3,4]

R$_f$ = 0.27 (cHex/ EtOAc 2:1).

2-((8-Hydroxynaphthalen-1-yl)oxy)isophthalonitrile 10

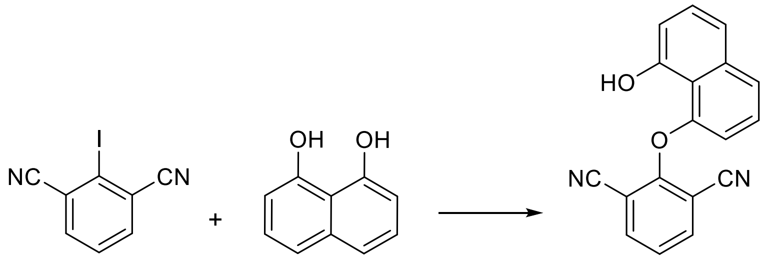

8 **9** **10**

NaH (0.38 g, 9.5 mmol, 1 eq, 60 % in mineral oil) was suspended in 4.5 mL of degassed DMF. Compound **9** (1.52 g, 9.5 mmol, 1 eq.) was dissolved in 12 mL of DMF, added to the mixture and stirred for 30 min. Subsequently compound **8** (2.41 g, 9.5 mmol, 1 eq.) was dissolved in 12 mL of DMF and added carefully to the mixture. The solution was heated to 90 °C and stirred for 6 h under strict exclusion of oxygen. After cooling down, 1 M HCl was added to the mixture and extracted with EtOAc. The organic phase was dried over Na_2SO_4 and the solvent was evaporated. The crude product was purified by column chromatography (EtOAc/ cHex 1:1) to yield **10** as a colorless foam (1.08 g, 40 %. Yield in other batches up to 62 %).

¹H-NMR (500 MHz, DMSO-*d*₆): δ [ppm] = 9.93 (s, 1H, OH), 8.15 (d, *J* = 7.8 Hz, 2H, Ar-H), 7.76 (dd, *J* = 8.3 + 0.9 Hz, 1H, Ar-H), 7.33 (m, 4H, Ar-H), 7.09 (dd, *J* = 7.6 + 0.9 Hz, 1H, Ar-H), 6.85 (dd, *J* = 7.3 + 1.0 Hz, 1H, Ar-H).

¹³C-NMR (126 MHz, DMSO-*d*₆): δ [ppm] = 160.30, 152.86, 150.66, 139.61, 136.96, 127.49, 126.40, 125.69, 123.79, 118.90, 117.41, 116.06, 114.39, 110.70, 103.98.

IR (cm⁻¹): 3503.07 (w), 2238.33 (w), 1611.50 (m), 1537.40 (m), 1450.48 (m), 1393.11 (s), 1243.80 (s), 1018.06 (m), 817.23 (m), 751.88 (s).

MS (ESI): m/z calcd. for $C_{18}H_{11}N_2O_2$ ([M+H⁺]): 287.07; found: 287.21.

HRMS: m/z calcd. for $C_{18}H_{11}N_2O_2$ ([M+H⁺]): 287.08150; found: 287.08142.

MP: 149-151°C Ref.: 149°C[5].

R_f = 0.44 (cHex/ EtOAc 1:1).

2-((8-Hydroxynaphthalen-1-yl)oxy)isophthalaldehyde 11

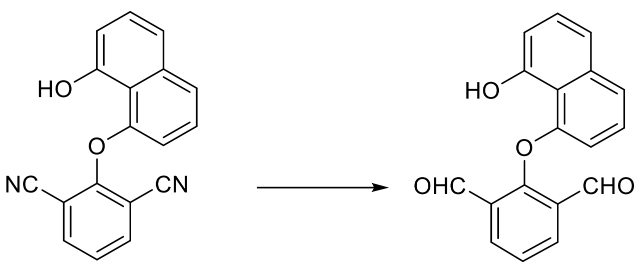

10 11

Compound **10** (860 mg, 3.0 mmol, 1 eq.) was dissolved in DCM (12.5 mL) and cooled to -78 °C. DIBAL-H (7.0 mL, 79.9 mmol, 3.3 eq., 20 wt% in toluene) was added carefully by use of a dropping funnel and stirred for 3.5 h at -78 °C. The mixture was warmed to 0 °C and 1 M HCl and DCM (30 mL each) were added. The suspension was stirred for 1 h at RT and extracted afterwards with DCM. The organic phase was dried over Na_2SO_4 and the solvent was evaporated. The crude product was purified by column chromatography (cHex/ EtOAc 5:1) to yield **11** as a yellow foam (407 mg, 46 %).

¹H-NMR (400 MHz, DMSO-*d*₆): δ [ppm] = 10.16 (s, 2H, 2x CHO), 9.68 (s, 1H, OH), 8.23 (*d*, *J* = 7.7 Hz, 2H, Ar-H), 7.70 (t, *J* = 7.7 Hz, 1H, Ar-H), 7.53 (d, *J* = 7.6 Hz, 1H, Ar-H), 7.46-7.38 (m, 2H, Ar-H), 7.22 (t, *J* = 8.0 Hz, 1H, Ar-H), 6.89 (m, 1H, Ar-H), 6.39 (d, *J* = 7.7 Hz, 1H, Ar-H).

¹³C-NMR (101 MHz, DMSO-*d*₆): δ [ppm] = 188.35, 158.04, 157.59, 153.85, 137.16, 135.15, 129.76, 127.89, 126.67, 125.90, 123.14, 118.79, 114.88, 111.28, 109.49.

IR (cm⁻¹): 3458.50 (m), 2923.24 (m), 2858.54 (m), 1683.43 (s), 1575.75 (s), 1451.19 (m), 1388.17 (s), 1298.91 (m), 1243.76 (m), 1197.72 (s), 1023.62 (s), 816.96 (s), 752.67 (s).

MS (ESI): m/z calcd. for $C_{18}H_{11}O_4$ ([M-H⁺]): 291.07; found: 291.36.

HRMS: m/z calcd. for $C_{18}H_{12}O_4$ ([M•+]): 292.07356; found: 292.07299.

R_f = 0.55 (EtOAc/cHex 2:1).

Boc-protected tetraaminonaphthol 12

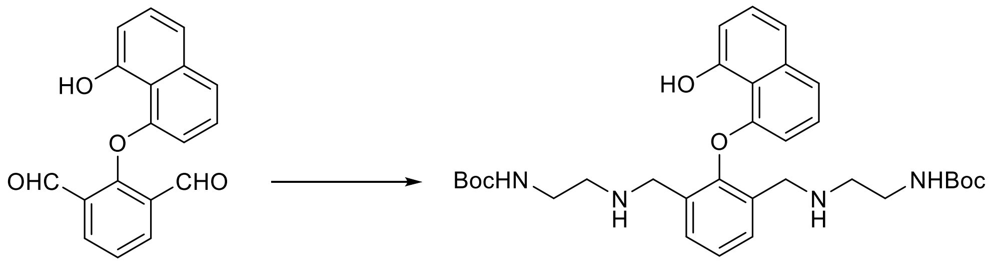

11 12

Compound **11** (200 mg, 0.68 mmol, 1 eq.) was dissolved in dry MeOH (7 mL) and *tert*-butyl(2-aminoethyl)carbamate **S1** (328 mg, 2.05 mmol, 3 eq.) was added. The solution was stirred for 4 h at RT and cooled down to 0 °C. $NaBH_4$ (155 mg, 4.11 mmol, 6 eq.) was added to the mixture in portions and stirred for 3 h at RT. The solvent was evaporated, and the residue was washed with NaCl (aq.) and extracted with EtOAc. The organic phase was dried over Na_2SO_4 and the solvent was removed under reduced pressure. After purification by column chromatography (DCM/ MeOH 20:1 + 1 % NEt_3) compound **12** was received as a colorless foam (362 mg, 91 %).

^{1}H-NMR (400 MHz, DMSO-*d$_6$*): δ [ppm] = 7.48-7.40 (m, 5H, Ar-H), 7.29 (t, *J* = 7.6 Hz, 1H, Ar-H), 7.20 (t, *J* = 8.0 Hz, 1H, Ar-H), 6.89 (dd, *J* = 6.5 + 2.2 Hz, 1H, Ar-H), 6.67 (t, *J* = 5.6 Hz, 2H), 6.21 (d, *J* = 7.7 Hz, 1H, Ar-H), 3.61 (d, *J* = 14.0 Hz, 2H, Ar-CH_2), 3.45 (d, *J* = 14.0 Hz, 2H, Ar-CH_2), 2.97-2.94 (m, 4H, CH_2), 2.42 (t, *J* = 6.5 Hz, 4H, CH_2), 1.34 (s, 18H, Boc-H).

^{13}C-NMR (101 MHz, DMSO-*d$_6$*): δ [ppm] = 155.54, 154.14, 153.92, 149.68, 136.96, 133.37, 128.79, 127.63, 125.88, 125.50, 121.84, 118.75, 115.04, 111.43, 106.26, 77.45, 48.61, 47.56, 28.22.

IR (cm^{-1}): 3349.29 (m), 2974.59 (m), 2930.22 (m), 1694.20 (s), 1513.14 (m), 1364.48 (m), 1248.45 (s), 1162.75 (s), 1028.49 (m), 817.09 (m), 755.05 (s).

MS (ESI): m/z calcd. for $C_{32}H_{43}N_4O_6$([M-H$^+$]): 579.32; found: 579.52.

HRMS: m/z calcd. for $C_{32}H_{45}N_4O_6$ ([M+H$^+$]): 581.33391; found: 581.33412.

R_f = 0.67 (DCM/ MeOH 20:1 + NEt_3).

8-(2,6-Bis(((2-aminoethyl)amino)methyl)phenoxy)naphthalen-1-ol 13

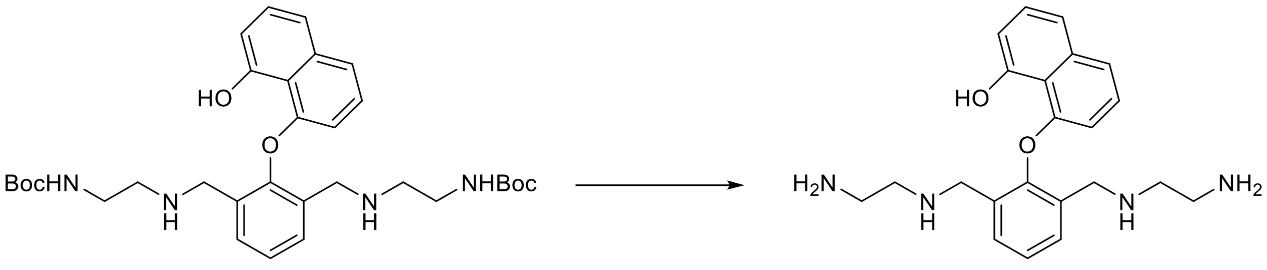

12 **13**

Compound **12** (45 mg, 0.077 mmol, 1 eq.) was dissolved in DCM (4 mL) and TFA (0.4 mL) was added. After stirring the solution for 45 min at RT, the product was precipitated by addition of Et_2O. The precipitate was filtered, washed with Et_2O and dried. The crude product was converted into the Cl-salt via ion exchange chromatography (Dowex® 1x8 Cl, MeOH) to yield **13** as a beige solid (28.7 mg, 71 %, calculated as tetra-hydrochloride).

^{1}H-NMR (600 MHz, DMSO-*d*$_6$): δ [ppm] = 10.10 (br, s, 2H, diastereotopic NH (tetrahydrochloride!)), 10.00 (br, s, 2H, diastereotopic NH'), 9.79 (br, s, 1H, OH), 8.37 (s, 6H, NH_3), 7.97 (d, *J* = 7.8 Hz, 2H, Ar-H), 7.58-7.54 (m, 2H, Ar-H), 7.44-7.41 (m, 2H, Ar-H), Ar-H, 7.22 (t, *J* = 8.0 Hz, 1H, Ar-H), 7.08 (m, 1H, Ar-H), 6.17 (d, *J* = 7.7 Hz, 1H, Ar-H), 4.14 (d, *J* = 13.6 Hz, 2H, Ar-CH_2), 3.98 (d, *J* = 13.5 Hz, 2H, Ar-CH_2), 3.22 (s, 6H, CH_2), 3.09 (s, 2H, CH_2), sample contains traces of MeOH.

^{13}C-NMR (126 MHz, DMSO-*d*$_6$): δ [ppm] = 154.12, 153.58, 150.92, 137.23, 133.02, 127.74, 126.54, 125.95, 125.82, 122.90, 118.97, 114.76, 111.59, 107.36, 48.57, 44.18, 43.58, 35.16, sample contains traces of MeOH.

IR (cm^{-1}): 2814.32 (w), 1575.98 (s), 1456.57 (m), 1194.03 (s), 1021.55 (s), 818.22 (s), 756.38 (s).

MS (ESI): m/z calcd. for $C_{22}H_{29}N_4O_2$([M+H$^+$]): 381.22; found: 381.31.

HRMS: m/z calcd. for $C_{22}H_{29}N_4O_2$ ([M+H$^+$]): 381.22850; found: 381.22863.

R_f = 0.62 (DCM/ MeOH 4:1 + NH_3).

tert-Butyl (2-aminoethyl)carbamate S1[6]

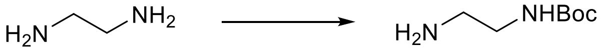

S1

Ethylene diamine (16.83 mL, 252 mmol, 10 eq.) was mixed with DCM (250 mL) and cooled to 0 °C. Boc_2O (5.5 g, 2.52 mmol, 1 eq) in 100 mL DCM was added slowly within 5 h via a dropping funnel. The mixture was stirred overnight and washed three times with water. The organic phase was dried over Na_2SO_4, and the solvent was evaporated to yield **S1** as a cloudy oil (3.52 g, 87 %).

^{1}H-NMR (400 MHz, DMSO-*d*$_6$): δ [ppm] = 6.73 (s, br, 1H, NH), 2.90 (q, *J* = 6.3 Hz, 2H, CH_2), 2.52 (t, *J* = 5.9 Hz, 2H, CH_2), 1,95 (s, br, 2H, NH_2), 1.37 (s, 9H, Boc-H).

^{13}C-NMR (101 MHz, DMSO-*d*$_6$): δ [ppm] = 155.66, 77.36, 43.60, 41.56, 28.23.

MS (ESI): m/z calcd. for $C_7H_{17}N_2O_2$([M+H$^+$]): 161.12; found: 161.18.

HRMS: m/z calcd. for $C_7H_{17}N_2O_2$ ([M+H$^+$]): 161.12845; found: 161.12824.

Boc-protected octaaminonaphthol 14

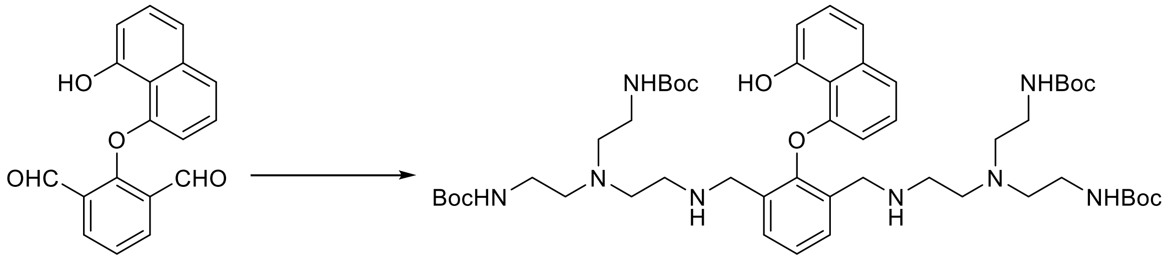

Compound **11** (50 mg, 0.17 mmol, 1 eq) was dissolved in dry MeOH (6 mL) and mixed with Di-Boc TREN **S2** (355 mg, 1.03 mmol, 6 eq.). The solution was heated to 55 °C and stirred for 6.5 h and overnight at RT. The mixture was cooled to 0 °C and $NaBH_4$ (40 mg, 1.03 mmol, 6 eq.) was added in portions. After stirring for 2 h at RT the solvent was evaporated. Brine was added to the residue, extracted with EtOAc and dried over Na_2SO_4. The solvent was removed under pressure and the crude product was purified by column chromatography (acetone/ cHex 2:1 + NH_3) to yield **14** as a colorless foam (57.6 mg, 35 %).

^{1}H-NMR (500 MHz, DMSO-*d*$_6$): δ[ppm] = 7.44 (m, 4H), 7.36 (m, 2H), 7.29 (t, *J* = 7.6 Hz, 1H), 7.19 (t, *J* = 8.0 Hz, 1H), 6.88 (dd, *J* = 6.6 + 2.1 Hz, 1H), 6.70 (br m, 4H), 6.23 (d, *J* = 7.0 Hz, 1H), 3.68 (d, *J* = 14.2 Hz, 2H), 3.50 (d, *J* = 14.2 Hz, 2H), 2.90 (br s, 8H), 2.44 (m, 4H), 2.37 (m, 12H), 1.30 (s, 36H).

^{13}C-NMR (126 MHz, DMSO-*d*$_6$): δ[ppm] = 155.66, 154.09, 153.98, 149.81, 137.00, 128.61, 127.61, 125.73, 125.50, 121.78, 118.60, 115.47, 111.71, 106.08, 77.37, 68.49, 55.82, 54.97, 54.08, 53.68, 53.46, 47.97, 46.78, 38.40, 32.09, 31.52, 29.52, 28.24, 28.18.

MS (ESI): m/z calcd. for $C_{50}H_{81}N_8O_{10}$ ([M+H$^+$]): 953.61; found: 954.18.

HRMS: m/z calcd. for $C_{50}H_{81}N_8O_{10}$ ([M+H$^+$]): 953.60702; found: 953.60651.

R$_f$ = 0.62 (acetone/ cHex 1:1 + 2 % NEt_3).

8-(2,6-Bis(((2-(bis(2-aminoethyl)amino)ethyl)amino)methyl)phenoxy) naphthalen-1-ol 15

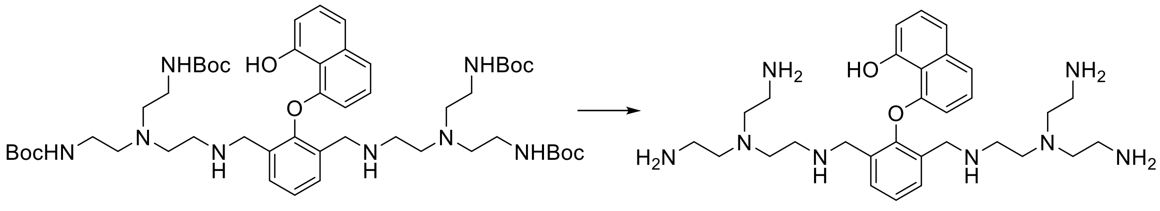

14 **15**

Compound **14** (38 mg, 0.04 mmol, 1 eq.) was dissolved in DCM (4 mL) and TFA (0.15 mL, 222 mg, 49 eq.) was added. The solution was stirred for 2.5 h at RT and the product precipitated afterwards with Et_2O. The precipitate was filtered, washed with Et_2O and dried. The crude product was converted into the Cl-salt by ion exchange chromatography (Dowex® 1x8 Cl^-, MeOH) to yield **15** as a colorless foam (23.1 mg, 75 %, calculated as hexahydrochloride).

^{1}H-NMR (500 MHz, DMSO-*d*$_6$): δ [ppm] = 9.67 (s, 1H, OH), 9.55 (s, br, 4H, NH), 8.12 (s, 12H), 8.02 (d, *J* = 7.8 Hz, 2H), 7.55 (d, *J* = 8.3 Hz, 1H), 7.51 (t, *J* =7.8 Hz, 1H), 7.44 (m, 2H), 7.23 (t, *J* = 8.0 Hz, 1H), 7.13 (m, 1H), 6.17 (d, *J* = 7.8 Hz, 1H), 4.15 (d, *J* = 13.0 Hz, 2H), 3.96 (d, *J* = 13.0 Hz, 2H), 3.19 (m, 2H), 3.13 (m, 2H), 3.01 (s, br, 8H), 2.78 (m, 4H), 2.64 (m, 8H), sample contains traces of MeOH.

^{13}C-NMR (126 MHz, DMSO-*d*$_6$): δ [ppm] = 154.13, 153. 49, 151.12, 137.15, 133.85, 127.97, 126.48, 125.95, 125.81, 123.02, 119.18, 114.70, 111.83, 107.23, 51.05, 49.80, 44.93, 44.82, 36.54, 29.59, sample contains traces of MeOH.

MS (ESI): m/z calcd. for $C_{30}H_{49}N_8O_2$ ([M+H$^+$]): 553.39; found: 553.45.

HRMS: m/z calcd. for $C_{30}H_{49}N_8O_2$ ([M+H$^+$]): 553.39730; found: 553.39660.

R$_f$ = 0 (acetone/ cHex 1:1 + 2 % NEt_3).

Di-Boc-TREN S2[7]

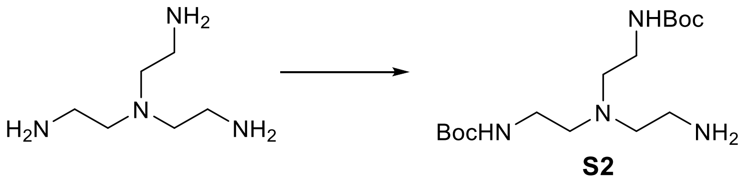

S2

TREN (1.39 g, 9.5 mmol, 1 eq.) was mixed with THF (40 mL) and cooled to 0 °C. Boc-ON (4.95 g, 20.1 mmol, 2.1 eq.) was dissolved in THF (50 mL) and added within 1 h via a dropping funnel. The solution was stirred overnight at RT and afterwards the solvent was evaporated. EtOAc was added to the residue and washed four times with 0.5 M NaOH. Brine was added to the aqueous phase, extracted with EtOAc and the combined organic phases were dried over Na_2SO_4. The solvent was removed under pressure and the crude product was purified by column chromatography (DCM/ MeOH 50:1 → 20:1 + 1 % NH_3) to yield **S2** as a brown oil (1.40 g, 44 %).

^{1}H-NMR (400 MHz, DMSO-*d*$_6$): δ [ppm] = 6.71 (m, 2H, NH_2), 2.97-2.92 (m, 4H), 2.47-2.53 (m, 2H, overlap with signal of DMSO), 2.43-2.38 (m, 6H), 1.37 (s, 18H, Boc-H).

^{13}**C-NMR (101 MHz, DMSO-*d*$_6$)**: δ [ppm] = 155.71, 77.43, 57.38, 54.03, 38.40, 28.24.

MS (ESI): m/z calcd. for $C_{16}H_{34}N_4O_4$ ([M+H$^+$]): 347.25; found: 347.60.

HRMS: m/z calcd. for $C_{16}H_{34}N_4O_4$ ([M+H$^+$]): 347.26528; found: 347.26549.

R$_f$ = 0.25 (DCM/MeOH/NH_3 20:1:0.04).

4-((5-(2,6-Bis(((2-((tert-butoxycarbonyl)amino)ethyl)amino)methyl)-phenoxy)-4-hydroxynaphthalen-1-yl)diazenyl)benzoic acid S3

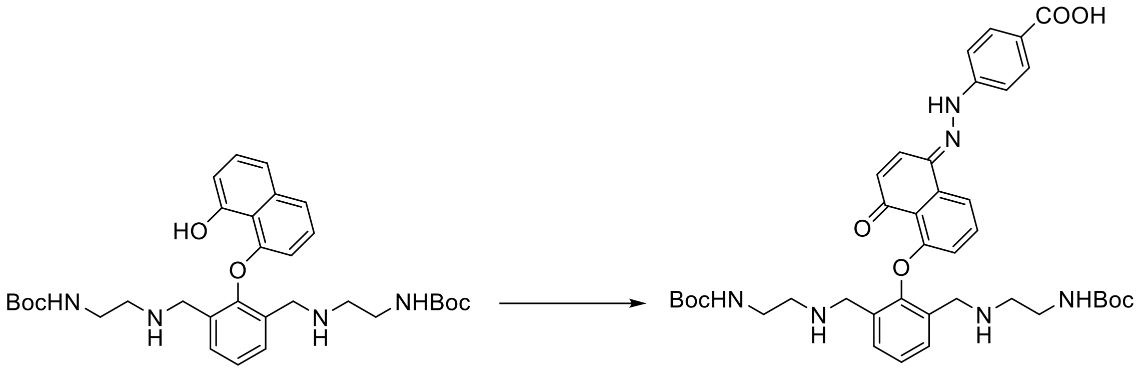

For the synthesis of the diazonium salt, 4-aminobenzoic acid (18.8 mg, 0.14 mmol, 1 eq.), was suspended with 6 M HCl (90 µL) and H_2O (50 µL) and cooled to 0 °C. $NaNO_2$ (10.4 mg, 0.15 mmol, 1.1 eq.) was dissolved in H_2O (200 µL) and added to the suspension. The mixture was stirred for 30 min at 0 °C.

Compound **12** (79.8 mg, 0.14 mmol, 1 eq.) and 2 M KOH in MeOH (500 µL) were mixed and cooled to 0 °C. Then the diazonium salt was added carefully to the solution, which directly turned violet. The reaction was stirred at 0-5 °C for 1 h and then diluted with brine. The solution was extracted with EtOAc and the solvent was evaporated. The crude product was purified by column chromatography (DCM/ MeOH 10:1 + NEt_3) to yield **S3** as an orange-red solid (46.1 mg, 46 %). The constitution of **S3** was derived from COSY, HSQC, and HMBC spectra.

^{1}**H-NMR (500 MHz, DMSO-*d*$_6$)**: δ [ppm] = 8.43 (d, *J* = 8.5 Hz, 1H), 8.07 (d, *J* = 9.1 Hz, 1H), 8.00 (d, *J* = 8.6 Hz 2H), 7.76 (d, *J* = 8.4 Hz, 2H), 7.53 (d, *J* = 7.6 Hz, 2H), 7.31 (m, 2H), 6.95 (m, br, 2H), 6.63 (d, *J* = 9.2 Hz, 1H), 6.26 (d, *J* = 7.6 Hz, 1H), 5.41 (s, br, 2H), 3.72 (d, *J* = 14.2 Hz, 2H), 3.61 (d, *J* = 14.2 Hz, 2H), 3.02-3.08 (m, 4H), 2.55-2.58 (m, 4H), 1.36 (s, 18H).

^{13}**C-NMR (126 MHz, DMSO-*d*$_6$)**: δ [ppm] = 172.07, 172.05, 168.74, 167.67, 161.74, 159.55, 159.52, 155.56, 151.68, 150.21, 146.65, 137.86, 137.27, 131.73, 131.58, 130.47, 129.73, 127.14, 125.49, 116.63, 116.21, 115.63, 77.58, 62.57, 57.55, 52.02, 48.14, 47.22, 33.27, 28.99, 28.21.

IR (cm^{-1}): 2921.2 (m), 2602.3 (m), 2496.0 (m), 1709.5 (w), 1475.7 (s), 1396.0 (s), 1172.8 (s), 1034.6 (s), 806.1 (m).

MS (ESI): m/z calcd. for $C_{39}H_{49}N_6O_8$ ([M+H$^+$]): 729.36; found: 729.22.

HRMS: m/z calcd. for $C_{39}H_{49}N_6O_8$ ([M+H$^+$]): 729.36064; found: 729.36009.

R$_f$ = 0.36 (DCM/ MeOH 10:1 + 2 % NEt_3)

Tetraamino azo naphthol 16

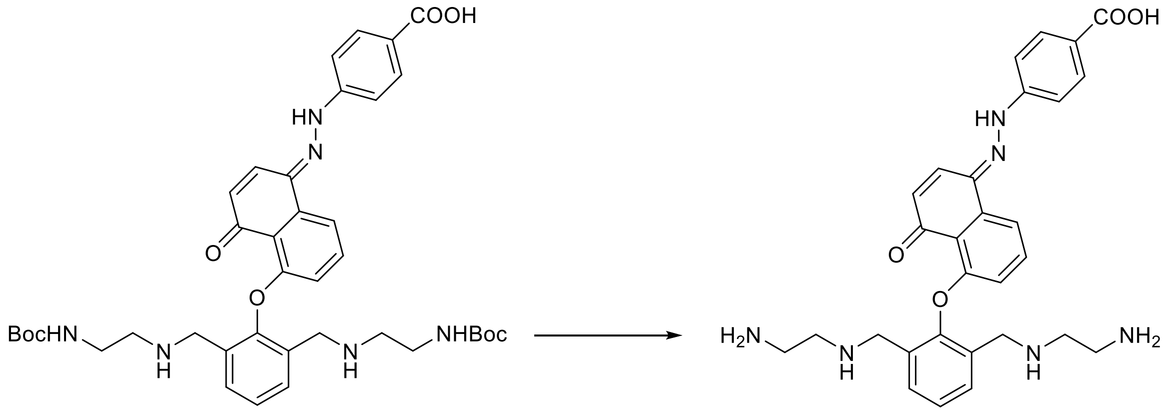

Compound **S3** (15 mg, 0.02 mmol, 1 eq.) was dissolved in a mixture of TFA/TIPS/H_2O (95/ 2.5/ 2.5) and stirred for 2h at RT. The solution was removed by a steam of argon. The crude product was converted into the Cl-salt via ion exchange chromatography (Dowex® 1x8 Cl, MeOH) to yield **16** as a dark red solid (11.4 mg, 84 %, calculated as tetrahydrochloride).

^{1}H-NMR (500 MHz, D_2O): δ [ppm] = 7.96 (d, *J* = 8.1 Hz, 1H), 7.81 (d, *J* = 10.2 Hz, 1H), 7.77 (d, *J* = 7.8 Hz, 2H), 7.57 (t, *J* = 7.8 Hz, 1H), 7.54 (d, *J* = 8.3 Hz, 2H), 7.45 (t, *J* = 8.2 Hz, 1H), 7.26 (d, *J* = 8.4 Hz, 2H), 6.62 (d, *J* = 10.2 Hz, 1H), 6.47 (d, *J* = 8.2 Hz, 1H), 4.26 (d, *J* = 14.0 Hz, 2H), 4.20 (d, *J* = 14.0 Hz, 2H), 3.42 (m, 8H).

^{13}C-NMR (126 MHz, D_2O): δ [ppm] = 187.44, 169.50, 155.81, 150.99, 146.76, 139.25, 134.24, 133.90, 132.07, 131.37, 128.76, 128.09, 125.81, 125.22, 123.70, 118.83, 118.53, 114.42, 112.32, 46.82, 44.24, 35.57.

IR (cm^{-1}): 2921.2 (m), 2602.3 (m), 2496.0 (m), 1709.5 (w), 1475.7 (s), 1396.0 (s), 1172.8 (s), 1034.6 (s), 806.1 (m).

MS (ESI): m/z calcd. for $C_{29}H_{33}N_6O_4$ ([M+H$^+$]): 529.25, found: 529.17.

HRMS: m/z calcd. for $C_{29}H_{33}N_6O_4$ ([M+H$^+$]): 529.25578; found: 529.25475.

R_f = 0 (DCM/ MeOH 4:1 + 2 % NH_3)

Di-tert-butyl ((((2-((8-hydroxy-5-((4-methoxyphenyl)diazenyl)naphthalen-1-yl)oxy)-1,3-phenylene)bis(methylene))bis(azanediyl))bis(ethane-2,1-diyl))-dicarbamate S4

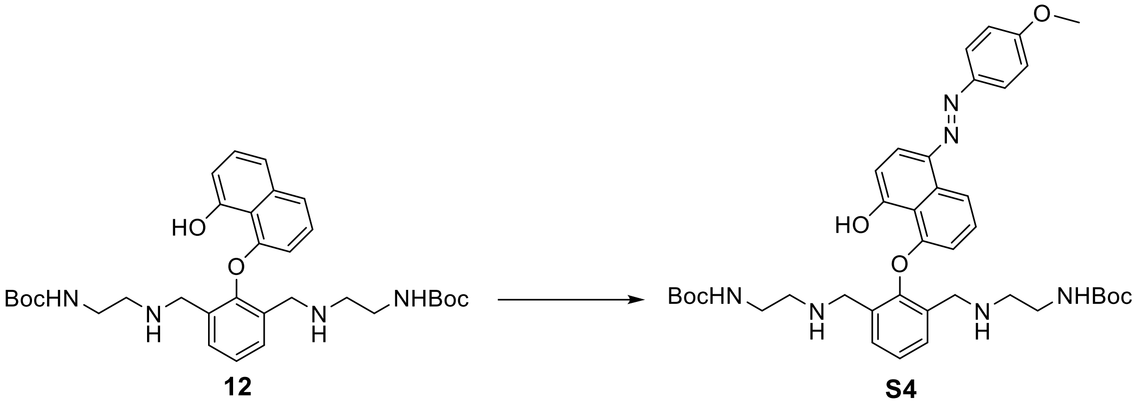

For the synthesis of the diazonium salt 4-methoxyaniline hydrochloride (24.6 mg, 0.15 mmol, 1 eq.) was suspended under argon with 6 M HCl (90 µL) and H_2O (60 µL) and cooled to 0 °C. $NaNO_2$ (11.7 mg, 0.17 mmol, 1.1 eq.) was dissolved in H_2O (300 µL) and added to the suspension. The mixture was stirred for 30 min at 0 °C.

Compound **12** (90 mg, 0.15 mmol, 1 eq.) and 2 M KOH in MeOH (600 µL) were mixed and cooled to 0 °C. Then the diazonium salt was added carefully to the suspension, which directly turned dark violet. The reaction was stirred at 0-5 °C for 2.5 h and then diluted with brine. The solution was extracted with DCM and the solvent was evaporated at RT. The crude product was purified by column chromatography (DCM/ MeOH 20:1 →10:1 + NH_3) to yield **S4** as a dark orange solid (38.6 mg, 36 %). The constitution of **S4** was derived from a COSY spectrum.

^{1}H-NMR (400 MHz, DMSO-*d*$_6$): δ [ppm] = 8.54 (d, *J* = 8.5 Hz, 1H), 7.93-7.89 (m, 3H), 7.53 (d, *J* = 7.7 Hz, 2H), 7.36 (m, 3H), 7.12 (d, *J* = 8.7 Hz, 2H), 6.91 (d, *J* = 8.6 Hz, 1H), 6.86-6.83 (m, 2H), 6.34 (d, *J* = 7.7 Hz, 1H), 3.87 (s, 3H), 3.72 (d, *J* = 14.0 Hz, 2H), 3.59 (d, *J* = 14.0 Hz, 2H), 3.03-3.01 (m, 4H), 2.51 (m, 4H, covered by DMSO), 1.36 (s, 18H).

^{13}C-NMR (101 MHz, DMSO-*d*$_6$): δ [ppm] = 161.15, 155.57, 158.27, 149.99, 135.60, 132.45, 129.45, 125.62, 123.94, 116.56, 114.55, 77.55, 69.78, 55.56, 48.38, 47.39, 28.21.

IR (cm^{-1}): 3374.8 (br, w), 2921.5 (s), 2854.4 (s), 1696.3 (m), 1457.0 (m), 1247.3 (s), 1029.0 (m),756.3 (m).

MS (ESI): m/z calcd. for $C_{39}H_{49}N_6O_7$ ([M-H$^+$]): 713.36; found: 713.38.

HRMS: m/z calcd. for $C_{39}H_{51}N_6O_7$ ([M+H$^+$]): 715.38137; found: 715.3808.

R$_f$ = 0.36 (DCM/ MeOH 10:1 + 2 % NEt_3)

Tetraamino azo naphthol 17

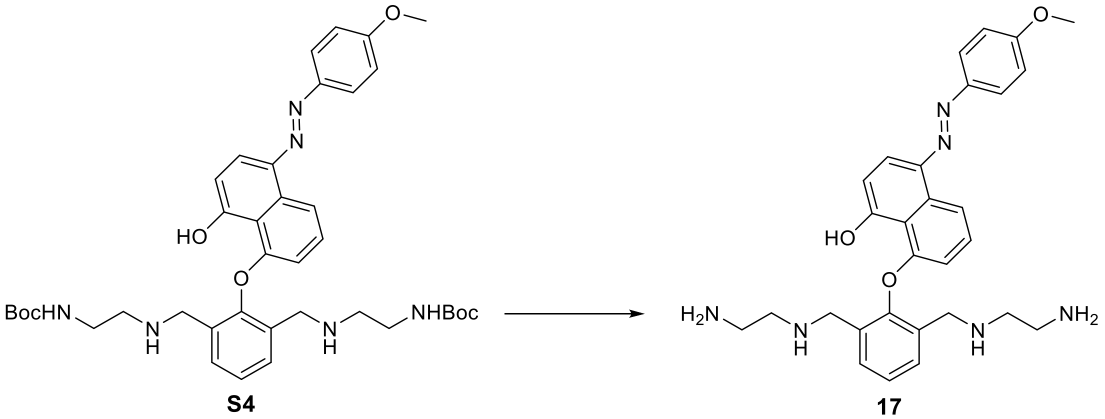

Compound **S4** (8.00 mg, 0.011 mmol, 1 eq.) was dissolved in a mixture of TFA/TIPS/H_2O (95/ 2.5/ 2.5; 1 mL) and stirred for 2h at RT. The solution was removed by a steam of argon. The crude product was converted into the Cl-salt via ion exchange chromatography (Dowex, MeOH) to yield **17** as a dark red solid (5.73 mg, 77 %, calculated as tetrahydrochloride).

^{1}H-NMR (400 MHz, D_2O): δ [ppm] = 8.47 (dd, *J* = 8.5, 0.9 Hz, 1H), 7.98 (d, *J* = 9.4 Hz, 1H), 7.84 (d, *J* = 7.8 Hz, 2H), 7.75 (d, *J* = 9.1 Hz, 2H), 7.64 (t, *J* = 7.8 Hz, 1H), 7.53 (t, *J* = 8.2 Hz, 1H), 7.09 (d, *J* = 9.1 Hz, 2H), 7.00 (d, *J* = 9.4 Hz, 1H), 6.55 (dd, *J* = 8.0, 0.9 Hz, 1H), 4.32 (d, *J* = 13.7 Hz, 2H), 4.27 (d, *J* = 13.7 Hz, 2H), 3.87 (s, 3H), 3.47 – 3.41 (m, 8H).

^{13}C-NMR (101 MHz, D_2O): δ [ppm] = 158.78, 154.87, 151.04, 137.52, 134.02, 130.80, 128.00, 125.28, 120.49, 118.42, 116.84, 114.94, 110.42, 55.68, 46.56, 44.18, 35.32.

IR (cm^{-1}): 3374.8 (br), 2924.9 (s), 2854.4 (m), 1729.8 (w), 1574.8 (m), 1456.7 (s), 1408.0 (s), 1240.0 (m), 1029.0 (m), 835.4 (w).

MS (ESI): m/z calcd. for $C_{29}H_{35}N_6O_3$ ([M+H$^+$]): 515.27, found: 515.09.

HRMS: m/z calcd. for $C_{29}H_{35}N_6O_3$ ([M+H$^+$]): 515.27652; found: 517.27590.

R_f = 0 (DCM/ MeOH 10:1 + 2 % NH_3)

Determination of pK_a Values

1 mL of a solution (approximately 50 µM) of compounds **13** or **16** in 100 mM phosphate buffer (NaH_2PO_4) was filled into a cuvette. First, two UV-Vis spectra were recorded at highly acidic and basic pH in the range from 200 to 800 nm. At wavelengths at which the difference in extinctions between the protonated and the deprotonated form were highest, the pKa values were determined by titrating from the acidic to the alkaline. For this purpose, a new 50 µM sample was prepared and titrated with NaOH. The pH was detected via a calibrated glass electrode. Plotting the extinction against pH values with Origin (OriginLab) led to the curves shown below. The p*K*a values are given by the points of inflection.

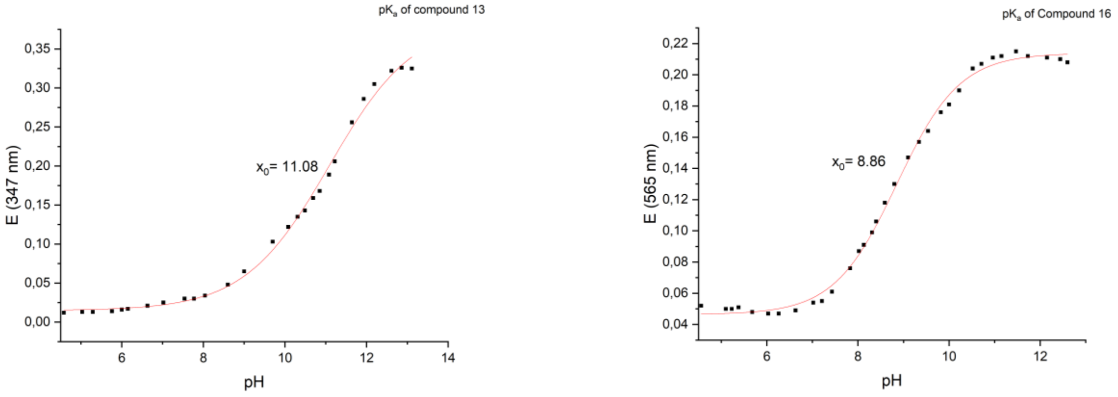

Figure S1. UV/VIS-spectrometric pK_a determination of compound **13** and **16**.

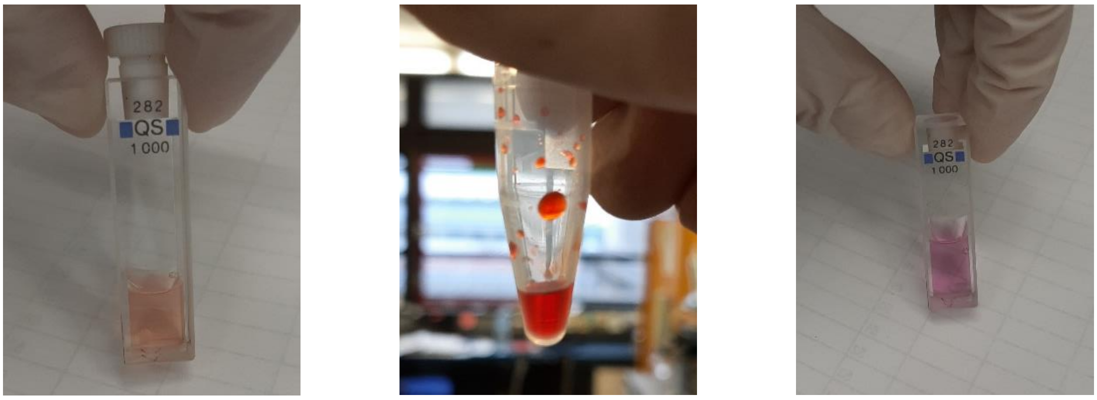

Figure S2. Color of compound **16** (H_2O solution) as a function of pH. Left: acidic; center: neutral; right: basic.

DNA Cleavage Assay

The pUC19 plasmid DNA, prepared from JM109 cells, was purified by using a NucleoBond® Xtra Midi Kit (Macherey-Nagel). The assay mixture (10 µL) contained (if not otherwise indicated) the hydrochlorides of compounds **13**, **16** or **17** at the given concentration in 50 mM HEPES-NaOH (pH 7.0 in most runs) and supercoiled pUC19 DNA (22.5 nM). Incubation was performed at 37 °C, typically for 20 h. Prior to electrophoresis, gel loading buffer (2 µL, 0.2 % crocein orange G, 40 % sucrose) and 20 % SDS (1 µL) were added to each sample. The addition of SDS and replacement of the usual tracking dye (bromophenol blue) with crocein orange G is recommended to prevent aggregation of compound and DNA during sample loading. Aliquots (10 µL) prepared in HEPES-buffer were loaded on a 1 % agarose gel that contained 0.5 µg mL^{-1} ethidium bromide (EtBr). TBE buffer (90 mM Tris, 90 mM boric acid, 2 mM EDTA) was used as the running buffer. After electrophoresis (120 V, 120 min), the gel was placed on a transilluminator (302 nm) and photographed through a yellow-orange filter (Etbr filter, Biostep). The digital photos were analyzed by using TotalLab Quant software (TotalLab). The peak areas of the supercoiled form, the open circle form and the linear form were determined, and the percentage open circle form was calculated. To normalize the decreased ability of EtBr to intercalate into form I DNA (relative to forms II and III) a factor of 1.22 was used.[1] The *nicked* control was prepared by cleaving pUC19 with the nicking endonuclease NB.BsrDI (New England Biolabs). For the *linearized* control, pUC19 was cleaved by the enzyme HindIII (New England Biolabs).

Samples for short-term kinetics (from 5 to 30 min, see Figure S7) were incubated directly before starting the electrophoresis. The samples were pipetted to the gel from the longest incubation time to the shortest and the electrophoresis was started immediately.

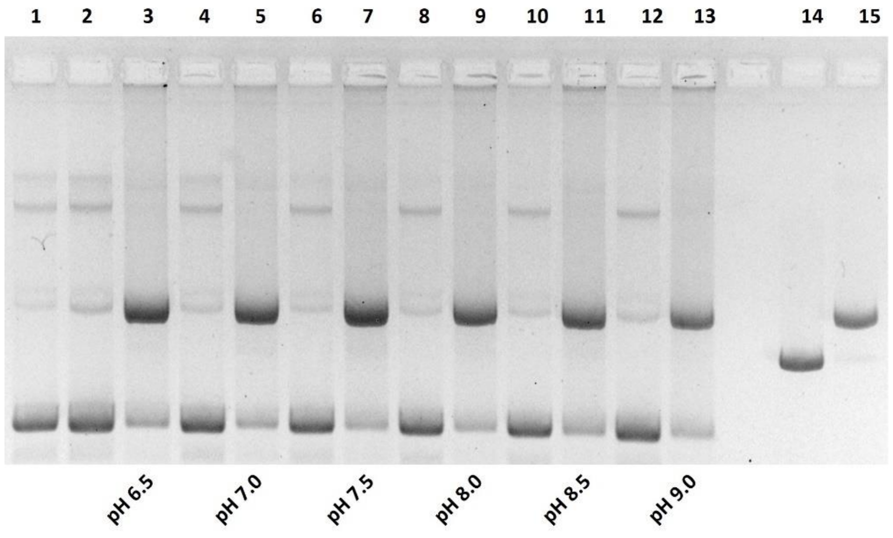

Figure S3. Cleavage of pUC19 by compound **13** (2 mM) as a function of pH. Lane 1 (from left): control 0 h; lane 2: control pH 6.5 20 h; lane 3: **13** pH 6.5; lane 4: control pH 7.0 20 h; lane 5: **13** pH 7.0; lane 6: control pH 7.5 20 h; lane 7: **13** pH 7.5; lane 8: control pH 8.0 20 h; lane 9: **13** pH 8.0; lane 10: control pH 8.5 20 h; lane 11: **13** pH 8.5; lane 12: control pH 9.0 20 h; lane 13: **13** pH 9.0; lane 14: linearized plasmid; lane 15: nicked plasmid (22.5 nM DNA, 50 mM HEPES NaOH, 37°C, 20 h; electrophoresis on 1% agarose gel, ethidium staining; inverted gray scale).

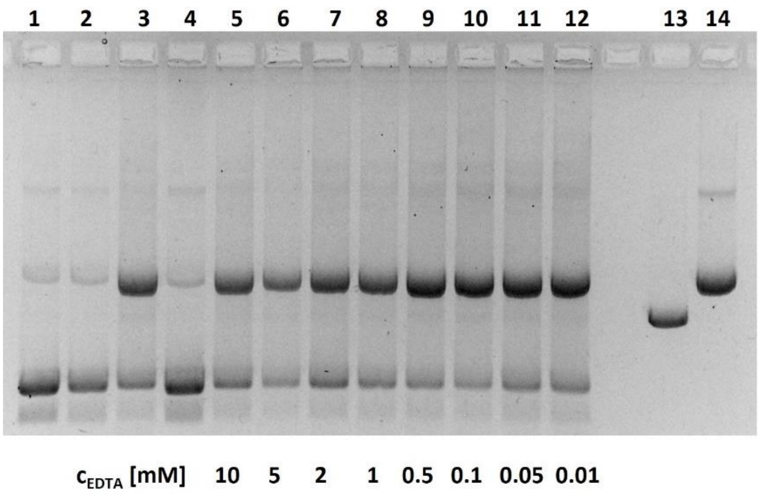

Figure S4. Cleavage of pUC19 by compound **13** (1 mM) in the presence of increasing concentrations of EDTA. Lane 1 (from left): control 0 h; lane 2: control 20 h; lane 3: control **13** 1mM 20 h; lane 4: control EDTA 20 h; lane 5: 10 mM EDTA; lane 6: 5 mM EDTA; lane 7: 2 mM EDTA; lane 8: 1 mM EDTA; lane 9: 0.5 mM EDTA; lane 10: 0.1 mM EDTA; lane 11: 0.05 mM EDTA, lane 12: 0.01 mM EDTA; lane 13: linearized plasmid; lane 14; nicked plasmid (22.5 nM DNA, 50 mM HEPES NaOH pH 7.0, 37 °C, 20 h, electrophoresis on 1% agarose gel, ethidium staining, inverted gray scale).

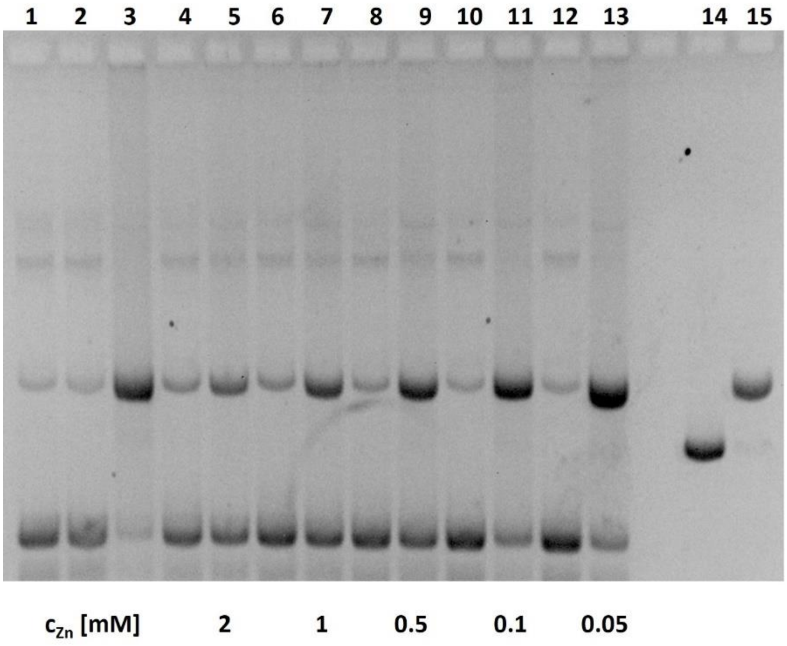

Figure S5. Cleavage of pUC19 by compound **13** (**4** mM) in the presence of increasing concentrations of Zn^{2+}. Lane 1 (from left): control 0 h; lane 2: control 20 h; lane 3: **13** control; lane 4: 2 mM Zn^{2+}; lane 5: **13** + 2 mM Zn^{2+}; lane 6: 1 mM Zn^{2+}; lane 7: **13** + 1 mM Zn^{2+}; lane 8: 0.5 mM Zn^{2+}; lane 9: **13** + 0.5 mM Zn^{2+}; lane 10: 0.1 mM Zn^{2+}; lane 11: **13** + 0.1 mM Zn^{2+}; lane 12: 0.05 mM Zn^{2+}; lane 13: **13** + 0.05 mM Zn^{2+}; lane 14: linearized plasmid; lane 15: nicked plasmid (22.5 nM DNA, 50 mM HEPES NaOH pH 7.0, 37°C, 20 h; electrophoresis on 1% agarose gel, ethidium staining; inverted gray scale).

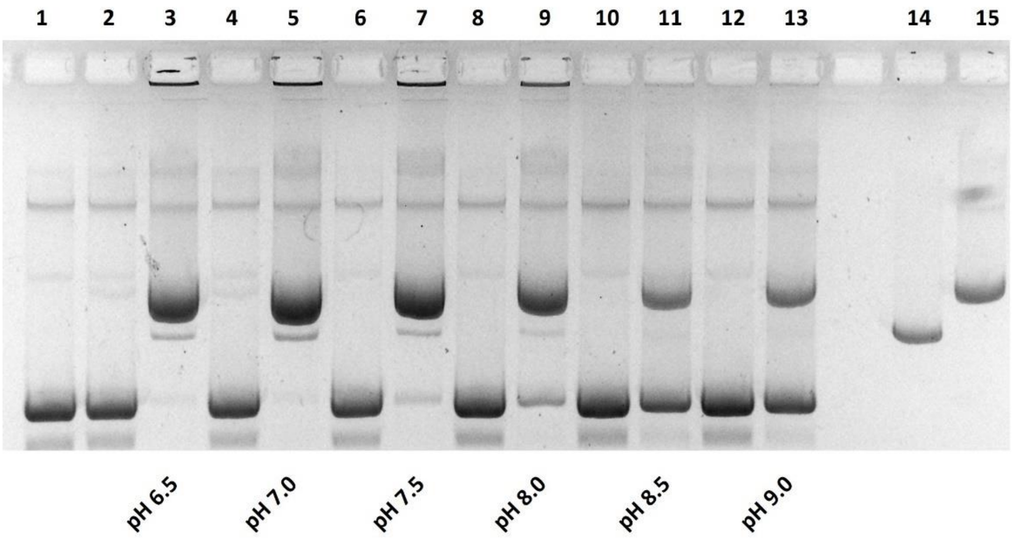

Figure S6. Cleavage of pUC19 by compound **16** (2 mM) as a function of pH. Lane 1 (from left): control 0 h; lane 2: control pH 6.5 20 h; lane 3: **16** pH 6.5; lane 4: control pH 7.0 20 h; lane 5: **16** pH 7.0; lane 6: control pH 7.5 20 h; lane 7: **16** pH 7.5; lane 8: control pH 8.0 20 h; lane 9: **16** pH 8.0; lane 10: control pH 8.5 20 h; lane 11: **16** pH 8.5; lane 12: control pH 9.0 20 h; lane 13: **16** pH 9.0; lane 14: linearized plasmid; lane 15: nicked plasmid (22.5 nM DNA, 50 mM HEPES NaOH, 37°C, 20 h; electrophoresis on 1% agarose gel, ethidium staining; inverted gray scale).

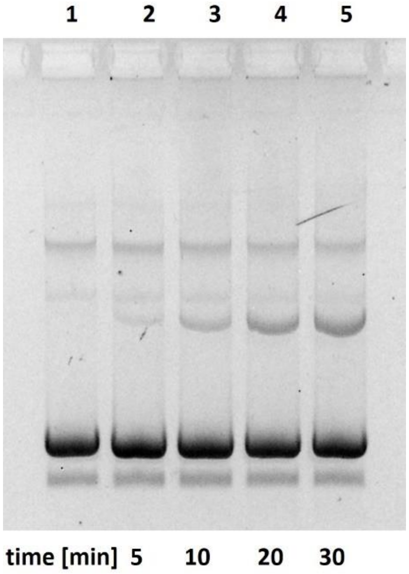

Figure S7. Short-term kinetics of pUC19 cleavage by 1 mM of **17**. Lane 1 (from left): control 0 h; lane 2: 5 min, lane 3: 10 min; lane 4: 20 min; lane 5: 30 min (22.5 nM DNA, 50 mM HEPES NaOH pH 7.0, 37 °C, 20 h, electrophoresis on 1% agarose gel, ethidium staining; inverted gray scale).

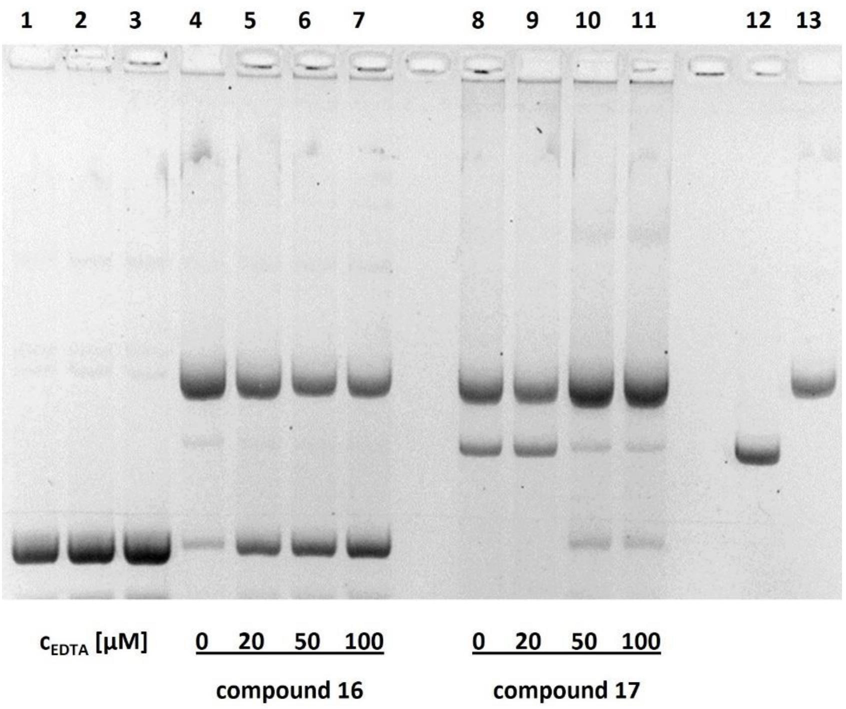

Figure S8. Influence of EDTA on pUC19 cleavage by 1 mM of compounds **16** and **17** (1 mM). Lane 1 (from left): control 0 h; lane 2: control 20 h; lane 3: control EDTA 100 µM; lane 4: control **16**, lane 5: **16** + 20 µM EDTA; lane 6: **16** + 50 µM EDTA; lane 7: **16** + 100 µM EDTA; lane 8: control **17**; lane 9: **17** + 20 µM EDTA; lane 10: **17** + 50 µM EDTA; lane 11: **17** + 100 µM EDTA; lane 12: linearized plasmid; lane 13: nicked plasmid (22.5 nM DNA, 50 mM HEPES NaOH pH 7.0, 37 °C, 20 h, electrophoresis on 1% agarose gel, ethidium staining; inverted gray scale).

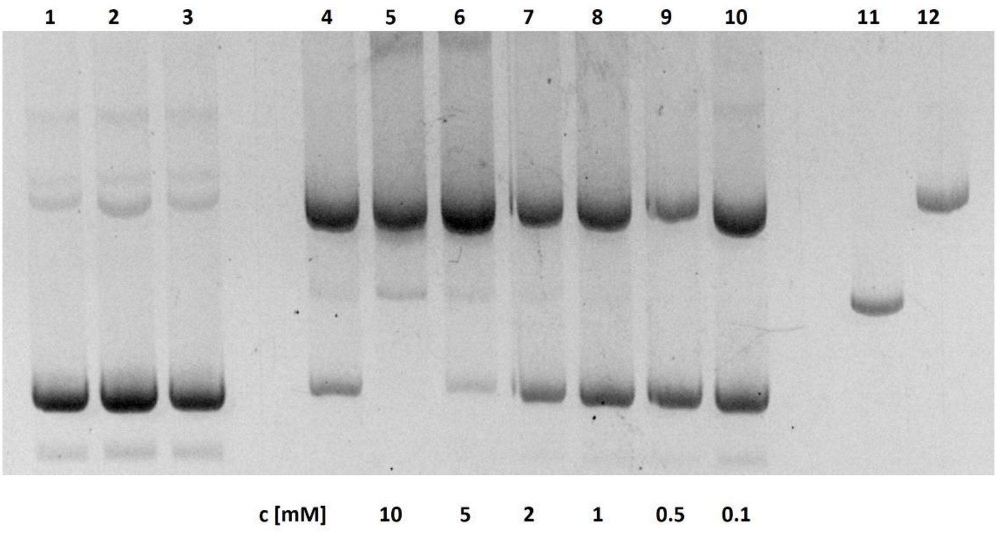

Figure S9. Influence of EDTA (0.5 mM) on pUC19 cleavage by 0.1-10 mM of compound **17**. Lane 1 (from left): control 0 h; lane 2: control 20 h; lane 3: control EDTA 0.5 mM; lane 4: control **17** 1 mM, lane 5: 10 mM **17** + 0.5 mM EDTA; lane 6: 5 mM **17** + 0.5 mM EDTA; lane 7: 2 mM **17** + 0.5 mM EDTA; lane 8: 1 mM **17** + 0.5 mM EDTA; lane 9: 0.5 mM **17** + 0.5 mM EDTA; lane 10: 0.1 mM **17** + 0.5 mM EDTA; lane 11: linearized plasmid; lane 12: nicked plasmid (22.5 nM DNA, 50 mM HEPES NaOH pH 7.0, 37 °C, 20 h, electrophoresis on 1% agarose gel, ethidium staining; inverted gray scale).

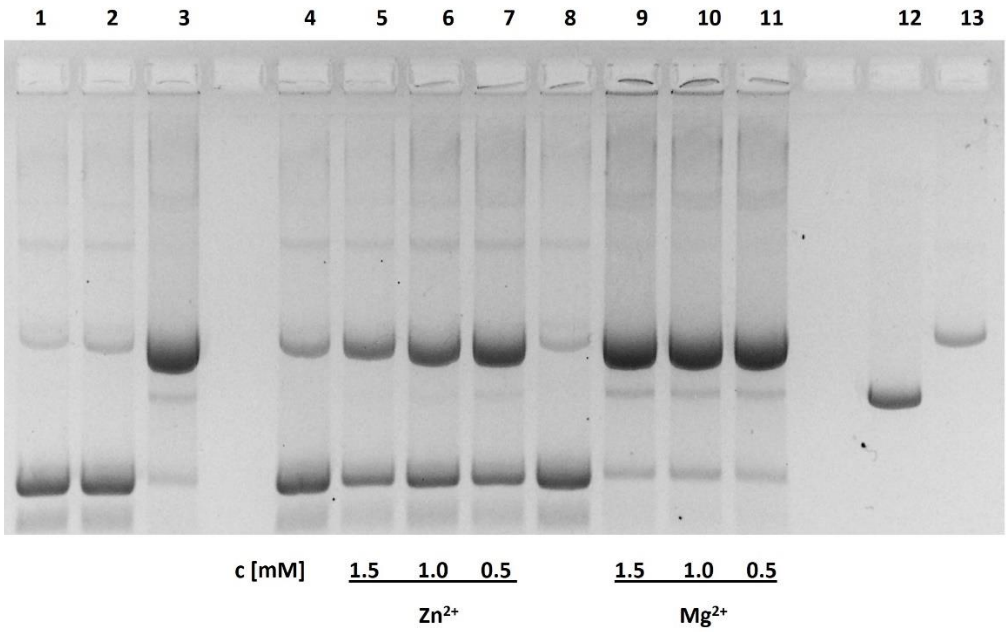

Figure S10. Influence of Zn^{2+} and Mg^{2+}on pUC19 cleavage by 1 mM of compound **17**. Lane 1 (from left): control 0 h; lane 2: control 20 h; lane 3: control **17** 20 h; lane 4: control 1.5 mM Zn^{2+}; lane 5: **17** + 1.5 mM Zn^{2+}; lane 6: **17** + 1.0 mM Zn^{2+}; lane 7: **17** + 0.5 mM Zn^{2+}; lane 8: control 1.5 mM Mg^{2+}; lane 9: **17** + 1.5 mM Mg^{2+}; lane 10: **17** + 1.0 mM Mg^{2+}; lane 11: **17** + 0.5 mM Mg^{2+}; lane 12: linearized plasmid; lane 13: nicked plasmid (22.5 nM DNA, 50 mM HEPES NaOH pH 7.0, 37 °C, 20 h, electrophoresis on 1% agarose gel, ethidium staining; inverted gray scale).

Kinetics

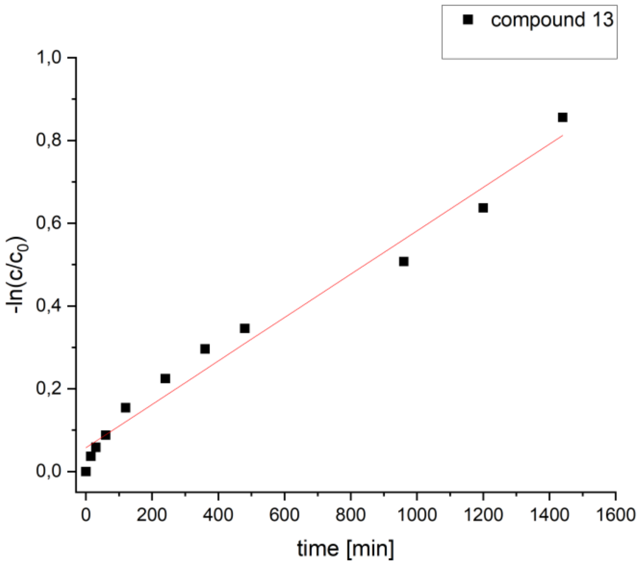

Figure S11. Cleavage kinetics of supercoiled pUC19 DNA by compound **13** (1 mM **13**, pH 7.0). Plot of $-\ln(c/c_0)$ vs reaction time (gel picture see Figure 3). With exception of the first data points, the reaction obeys first-order kinetics. Slope of the regression line: $5.24 \pm 0.27 \times 10^{-4}$ min^{-1}, t ½ = 22 h.

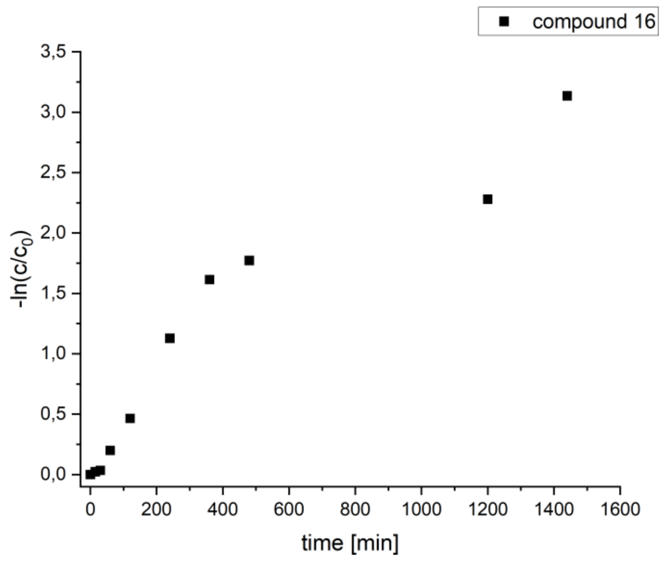

Figure S12. Cleavage kinetics of supercoiled pUC19 DNA by compound **16** (1 mM **16**, pH 7.0). Plot of -ln(c/c_0) vs reaction time (gel picture see Figure 6). Data does not allow to draw a meaningful regression line. Nevertheless, about 50 % of the plasmid is cleaved within the first 3 h.

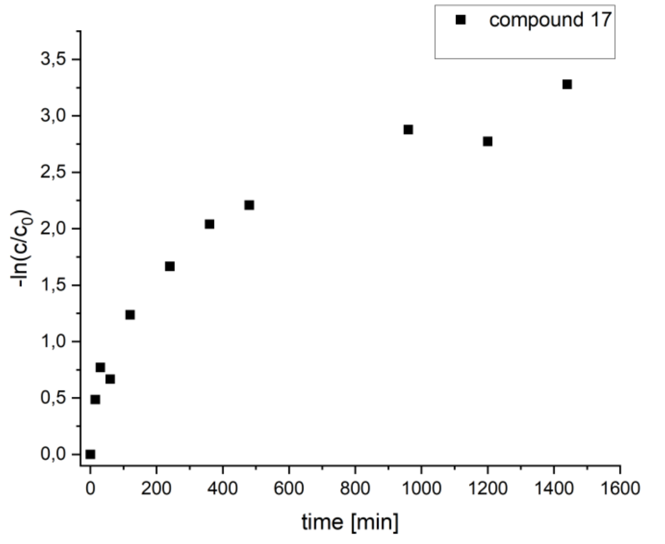

Figure S13. Cleavage kinetics of supercoiled pUC19 DNA by compound **17** (1 mM **17**, pH 7.0). Plot of -ln(c/c_0) vs reaction time (gel picture see Figure 8). The reaction does not follow first-order kinetics but slows down with time. Nevertheless, about 50 % of the plasmid is cleaved within the first hour.

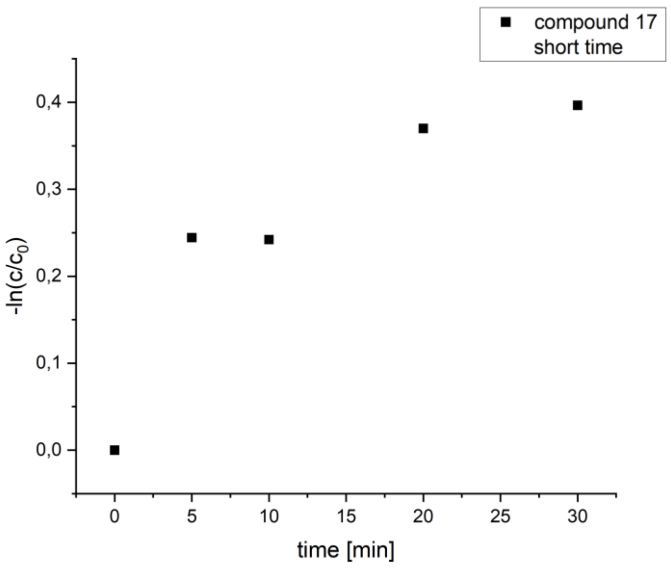

Figure S14. Short-time kinetics with compound **17** (1 mM **17**, pH 7.0). Plot of -ln(c/c_0) vs reaction time (gel picture see Figure S7).

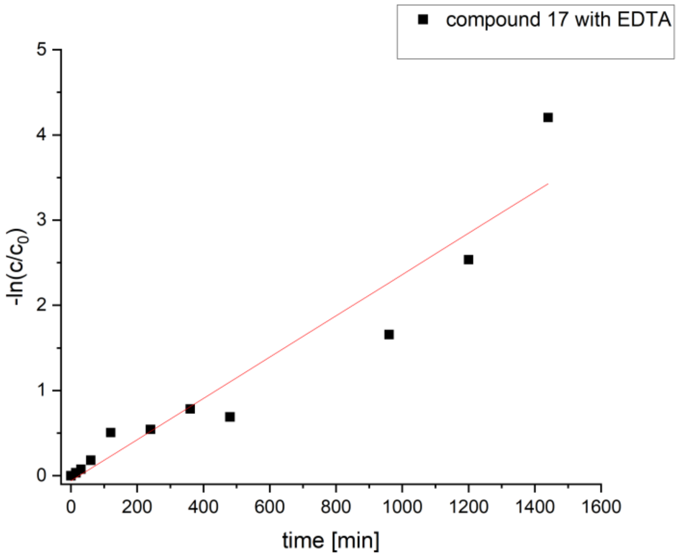

Figure S15. Cleavage kinetics of supercoiled pUC19 DNA by compound **17** in the presence of EDTA (1 mM **17**, 0.1 mM EDTA, pH 7.0). Plot of -ln(c/c_0) vs reaction time (gel picture see Figure 9). The reaction follows first-order kinetics fairly well. Slope of the regression line: 2.42 ± 0.24 x 10^{-3} min^{-1}, t ½ = 4.8 h.

XRF Measurements

The XRF data were recorded by Prof. Dr. Nele Meyer (Institute for Physical Geography, Goethe Universität Frankfurt) with a Niton™ XL3t GOLDD+ XRF Analyzer (Thermo Fisher). The samples were placed in a XRF sample cup SC-4331 (Fisher Scientific) and covered with a polypropylene XRF Film Roll TF-240 (Fisher Scientific). 50 mg of **13** and 30 mg of **17** were used for the experiments.

NMR Spectra

Compound 8

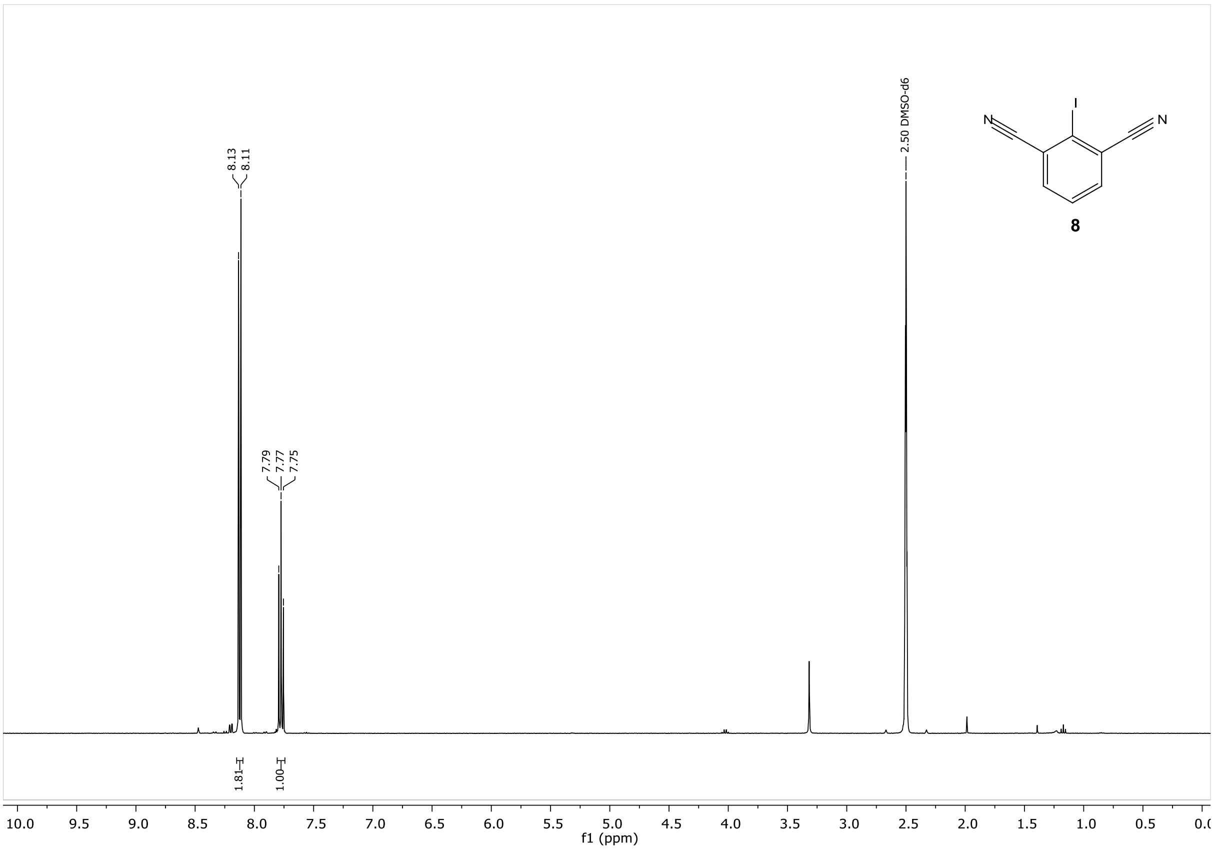

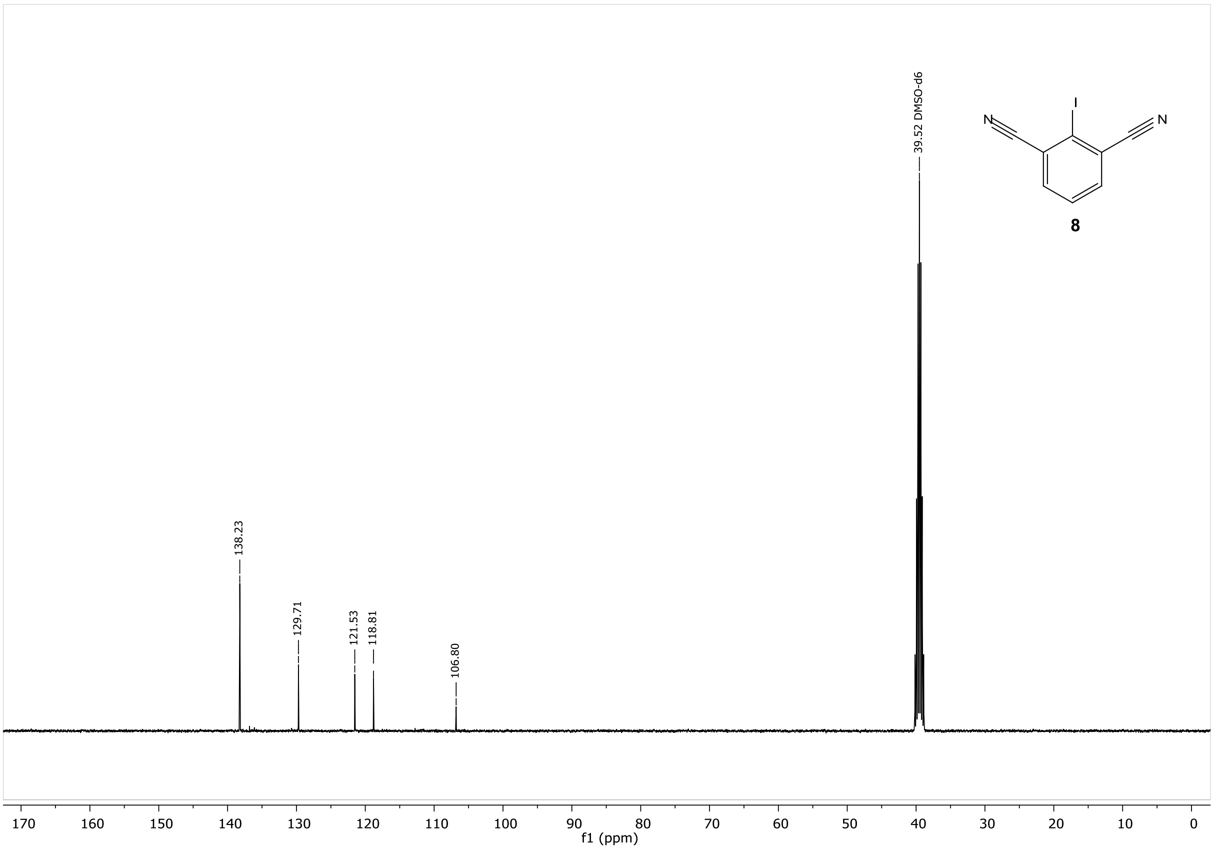

Compound 9

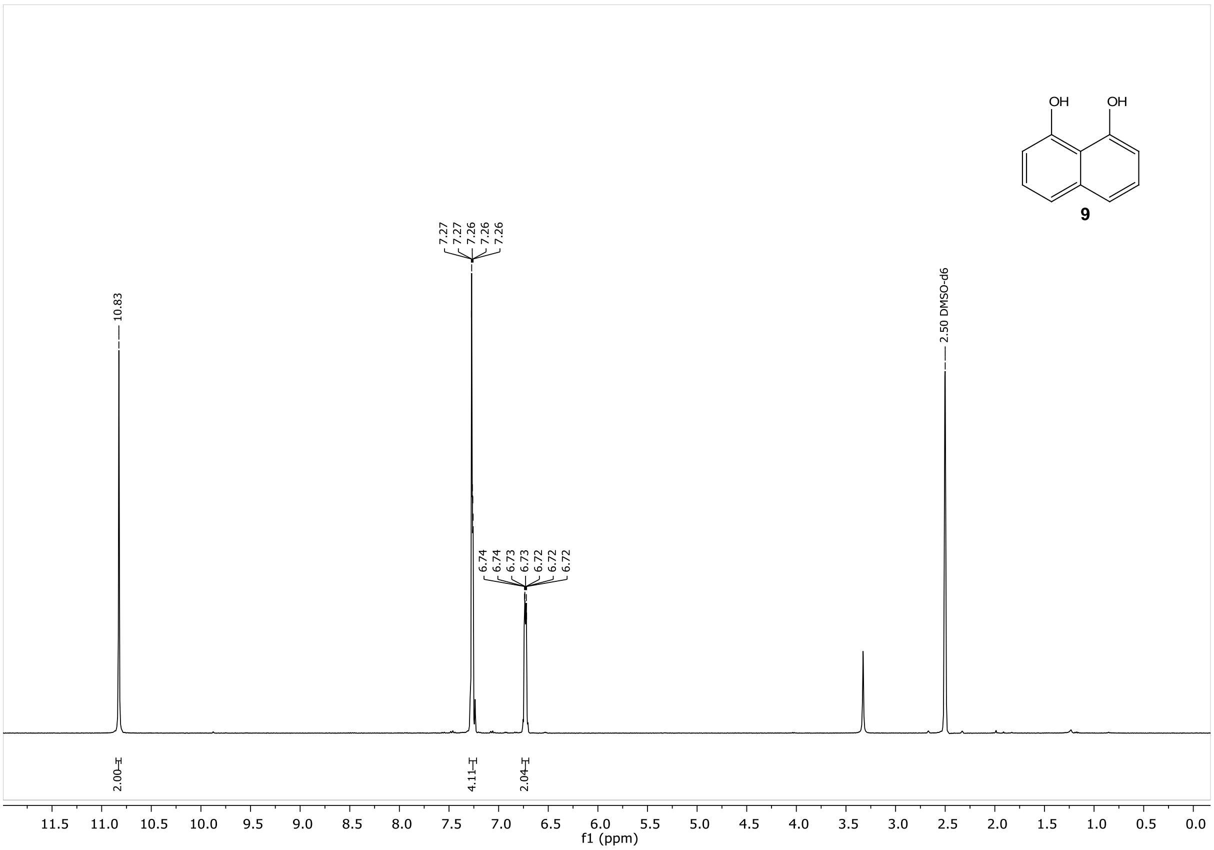
OH OH
9
7.27
7.27
7.26
7.26
7.26
7.26
10.83
2.50 DMSO-d6
6.74
6.74
6.73
6.73
6.72
6.72
6.72
2.00
4.11
2.04
11.5 11.0 10.5 10.0 9.5 9.0 8.5 8.0 7.5 7.0 6.5 6.0 5.5 5.0 4.5 4.0 3.5 3.0 2.5 2.0 1.5 1.0 0.5 0.0
f1 (ppm)

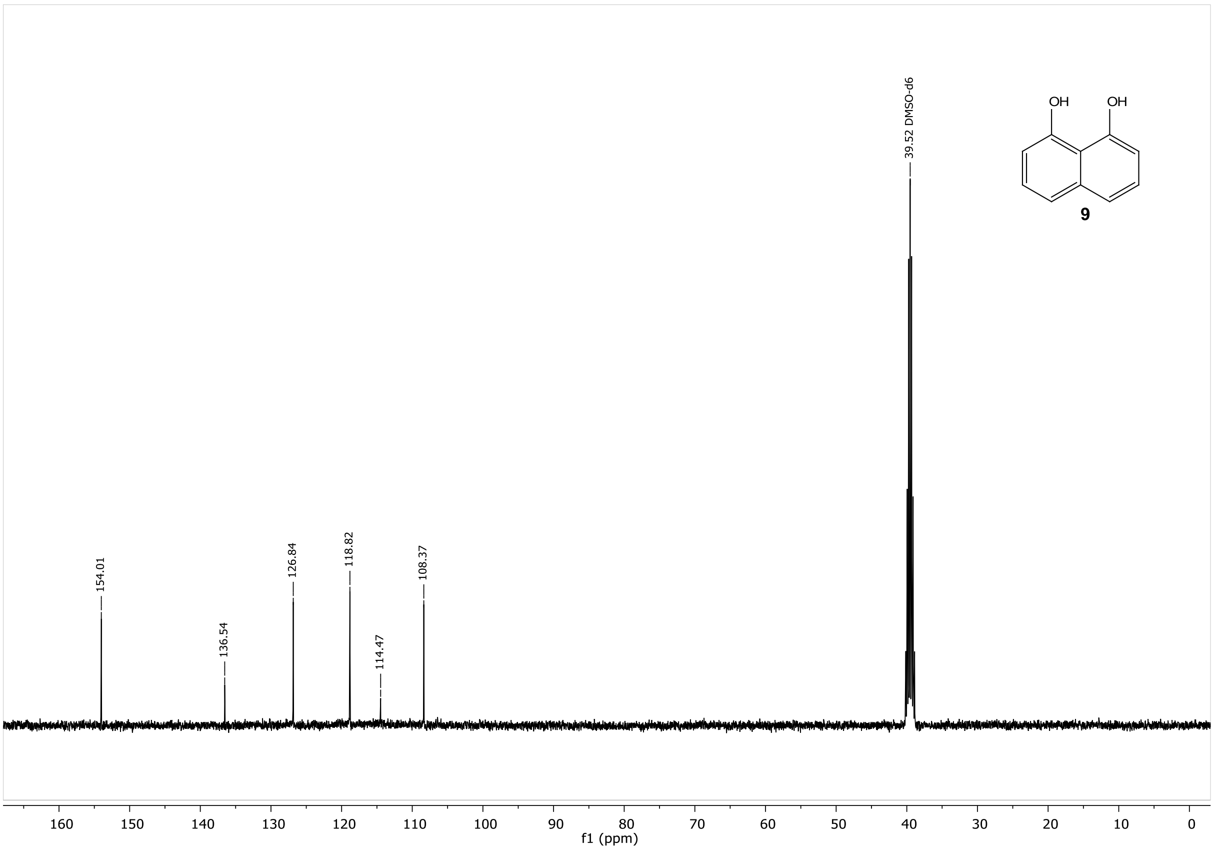
39.52 DMSO-d6
OH OH
9
154.01
136.54
126.84
118.82
114.47
108.37
160 150 140 130 120 110 100 90 80 70 60 50 40 30 20 10 0
f1 (ppm)

Compound 10

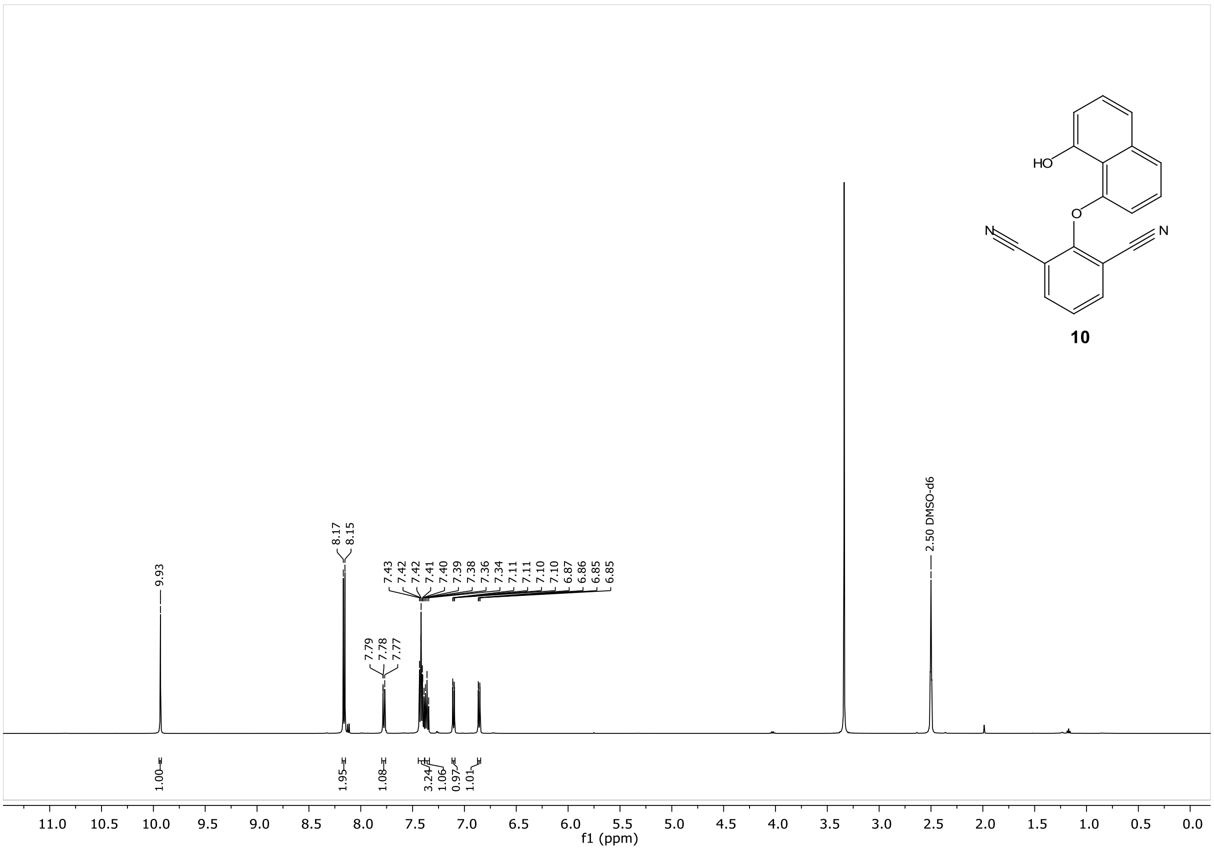

HO
O
N
N
10
9.93
8.17
8.15
7.79
7.78
7.77
7.43
7.42
7.42
7.41
7.40
7.39
7.38
7.36
7.34
7.11
7.11
7.11
7.10
7.10
6.87
6.86
6.85
6.85
2.50 DMSO-d6
1.00
1.95
1.08
3.24
1.06
0.97
1.01
f1 (ppm)

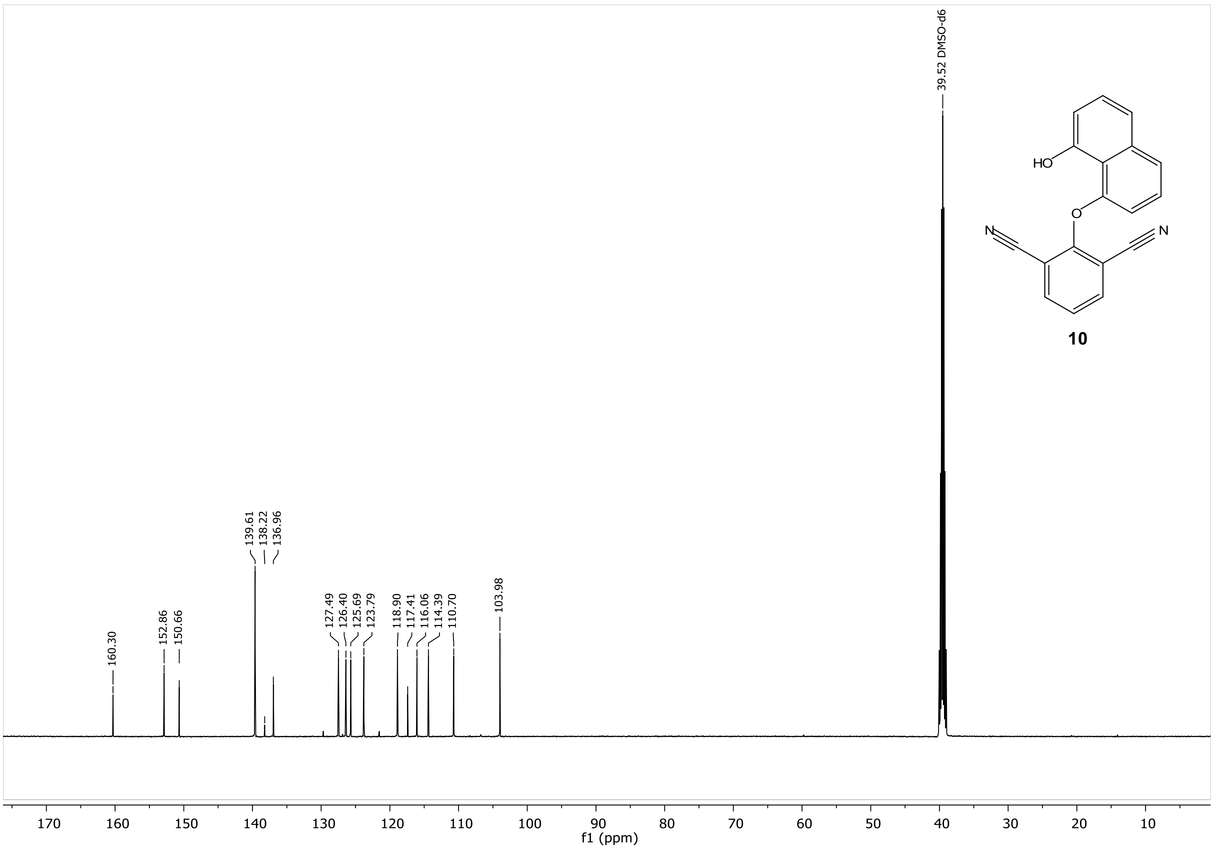

HO
O
N
N
10
39.52 DMSO-d6
160.30
152.86
150.66
139.61
138.22
136.96
127.49
126.40
125.69
123.79
118.90
117.41
116.06
114.39
110.70
103.98
f1 (ppm)

Compound 11

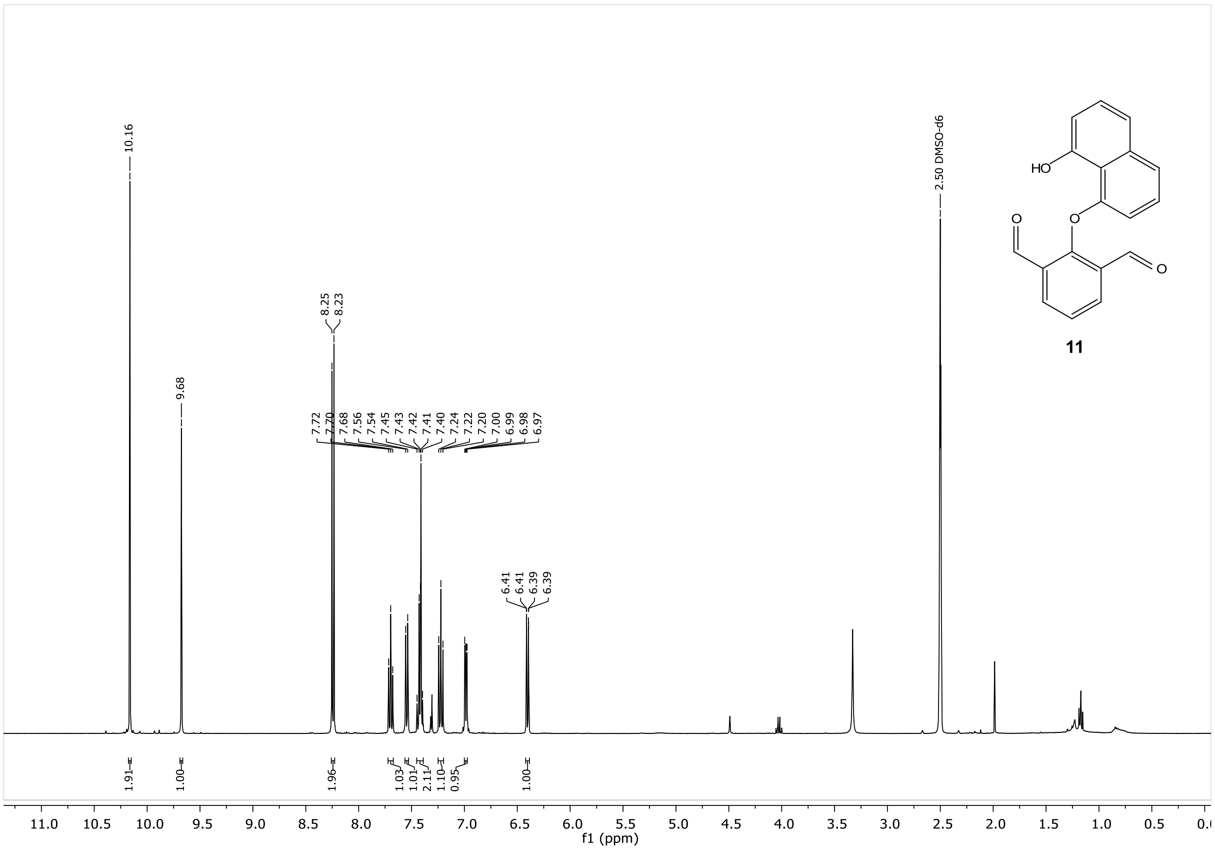
10.16
9.68
8.25
8.23
7.72 7.70 7.68 7.56 7.54 7.45 7.43 7.42 7.41 7.40 7.24 7.22 7.20 7.00 6.99 6.98 6.97
6.41 6.41 6.39 6.39
2.50 DMSO-d6
HO
O
O
O
11
1.91
1.00
1.96
1.03
1.01
2.11
1.10
0.95
1.00
f1 (ppm)

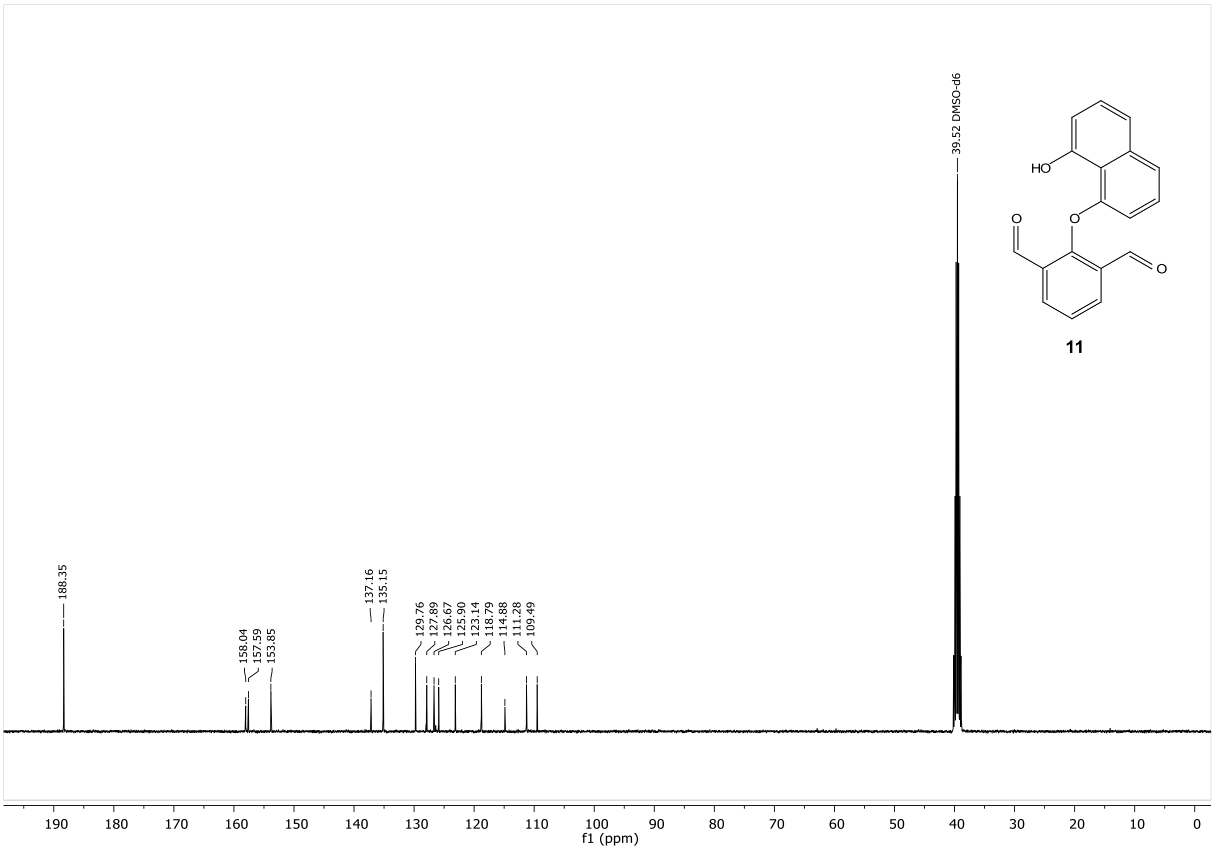
188.35
158.04
157.59
153.85
137.16
135.15
129.76 127.89 126.67 125.90 123.14 118.79 114.88 111.28 109.49
39.52 DMSO-d6
HO
O
O
O
11
f1 (ppm)

Compound S1

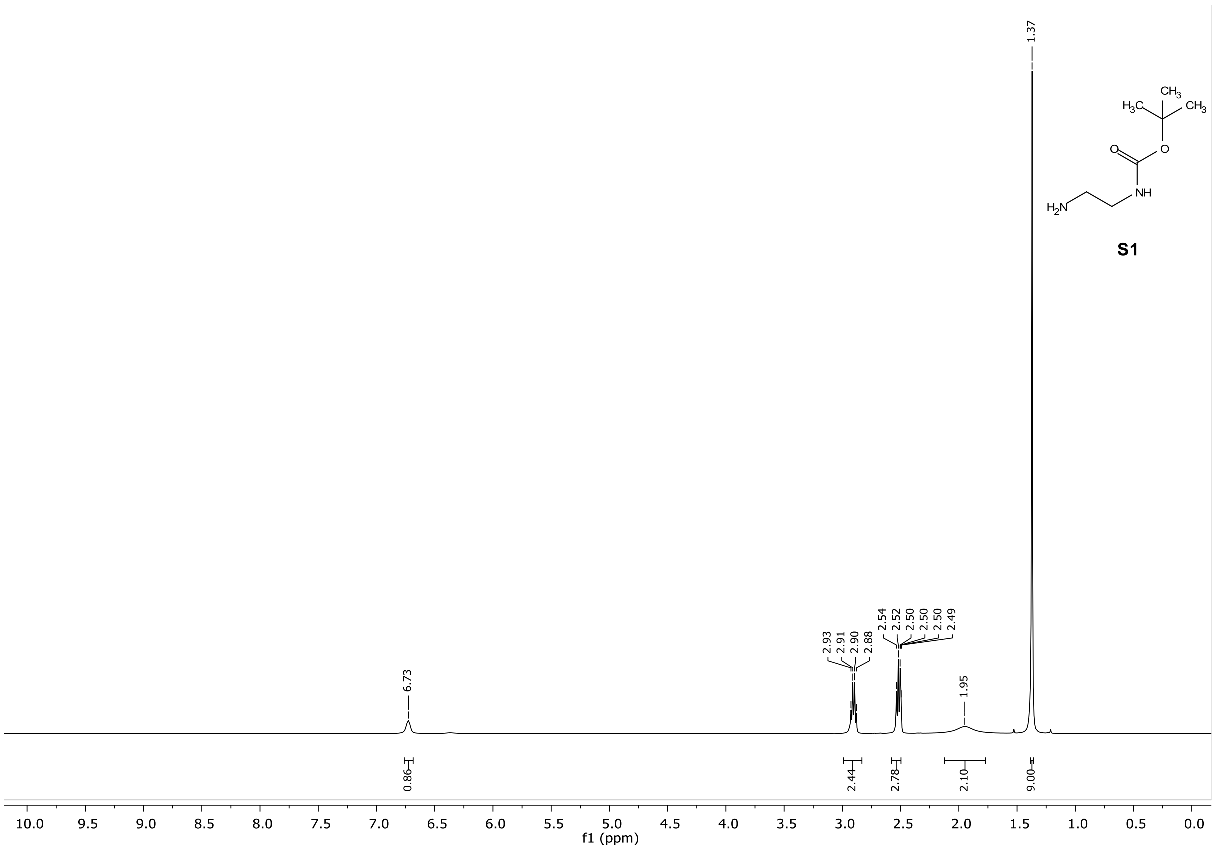

1.37
6.73
2.93
2.91
2.90
2.88
2.54
2.52
2.50
2.50
2.50
2.50
2.49
1.95
0.86
2.44
2.78
2.10
9.00
f1 (ppm)
S1

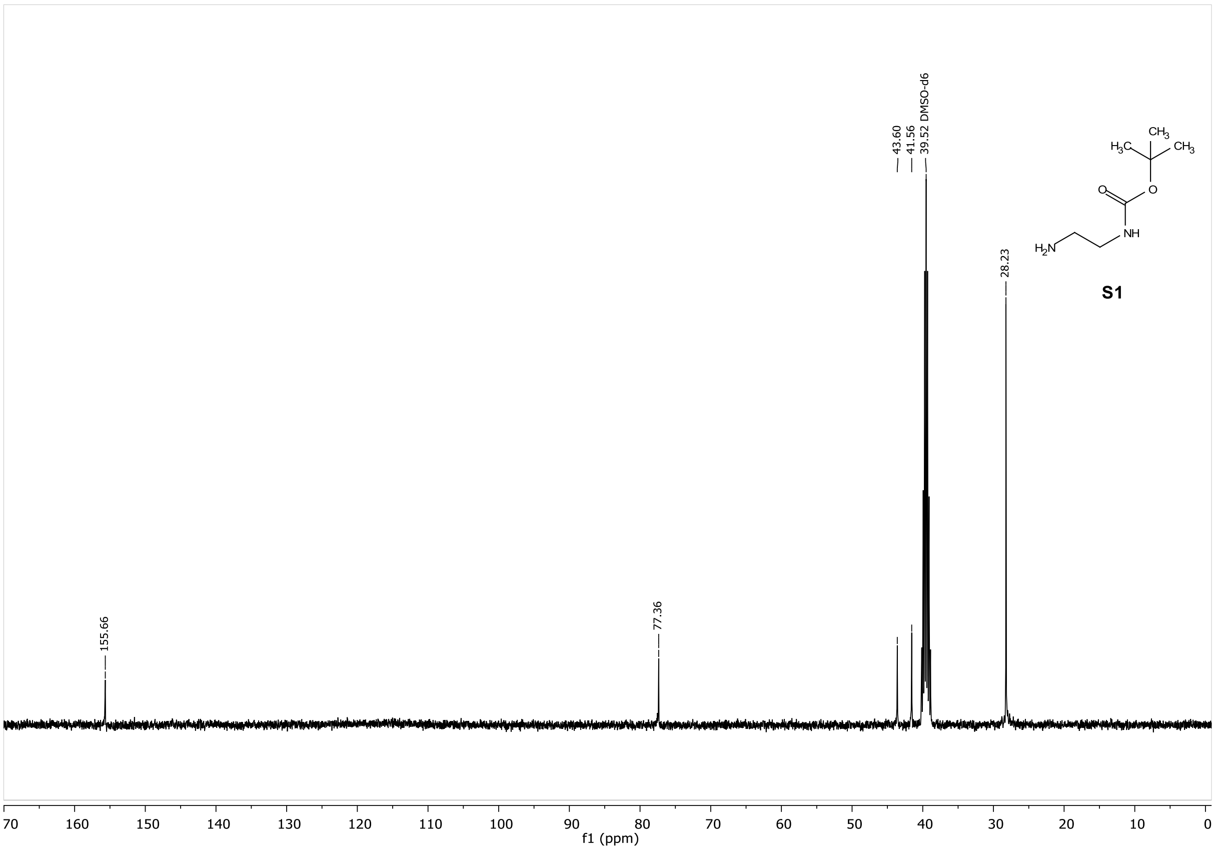

155.66
77.36
43.60
41.56
39.52 DMSO-d6
28.23
f1 (ppm)
S1

Compound 12

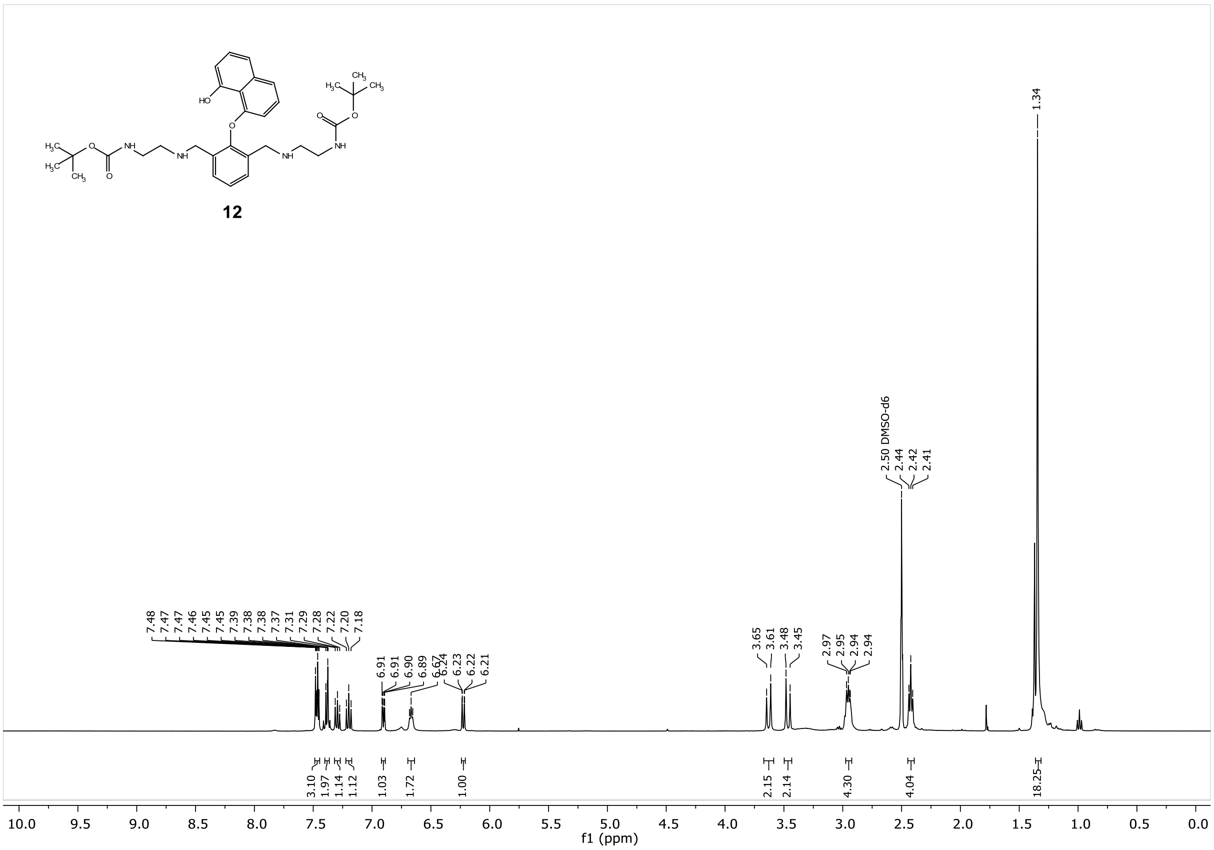
12
1.34
2.50 DMSO-d6
2.44
2.42
2.41
7.48
7.47
7.47
7.46
7.45
7.45
7.39
7.38
7.38
7.37
7.31
7.29
7.28
7.22
7.20
7.18
6.91
6.91
6.90
6.89
6.67
6.24
6.23
6.22
6.21
3.65
3.61
3.48
3.45
2.97
2.95
2.94
2.94
3.10
1.97
1.14
1.12
1.03
1.72
1.00
2.15
2.14
4.30
4.04
18.25
f1 (ppm)

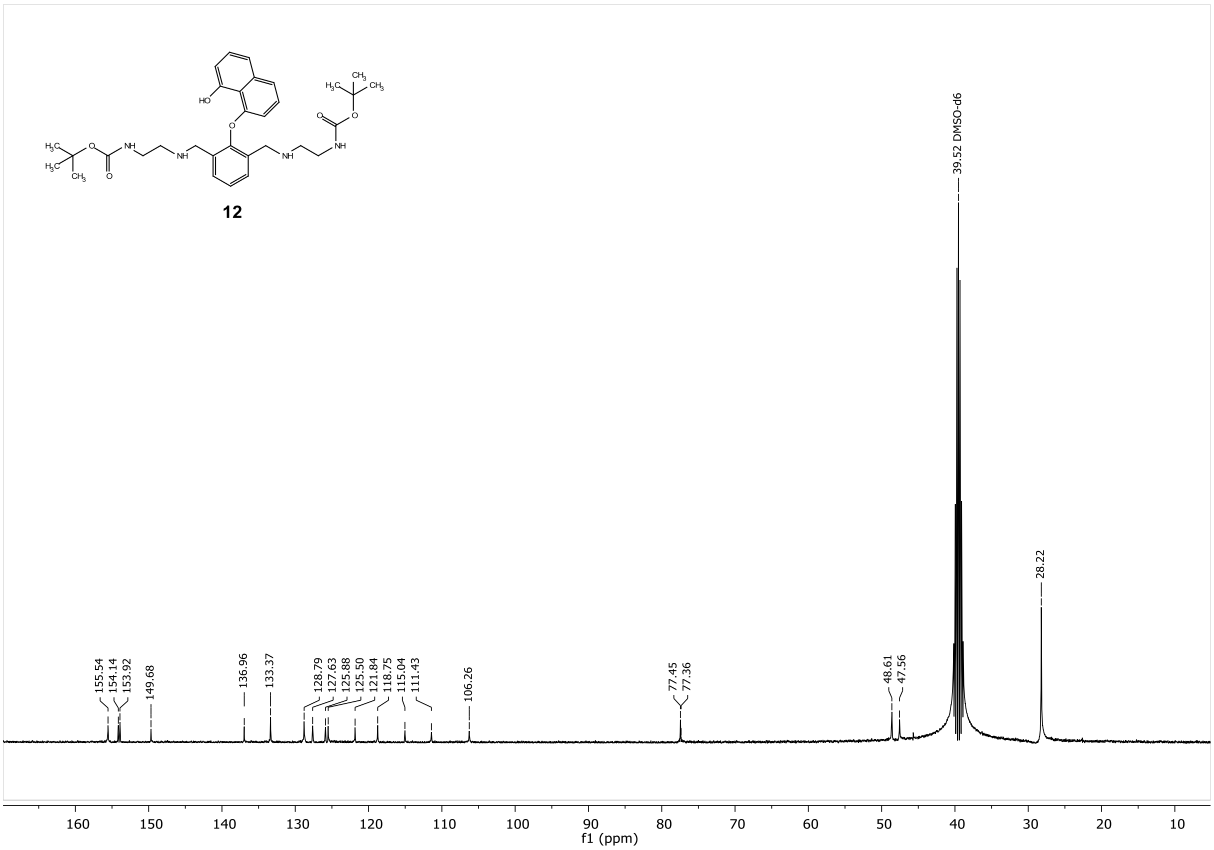
12
39.52 DMSO-d6
28.22
155.54
154.14
153.92
149.68
136.96
133.37
128.79
127.63
125.88
125.50
121.84
118.75
115.04
111.43
106.26
77.45
77.36
48.61
47.56
f1 (ppm)

Compound 13 as tetrahyddrochloride

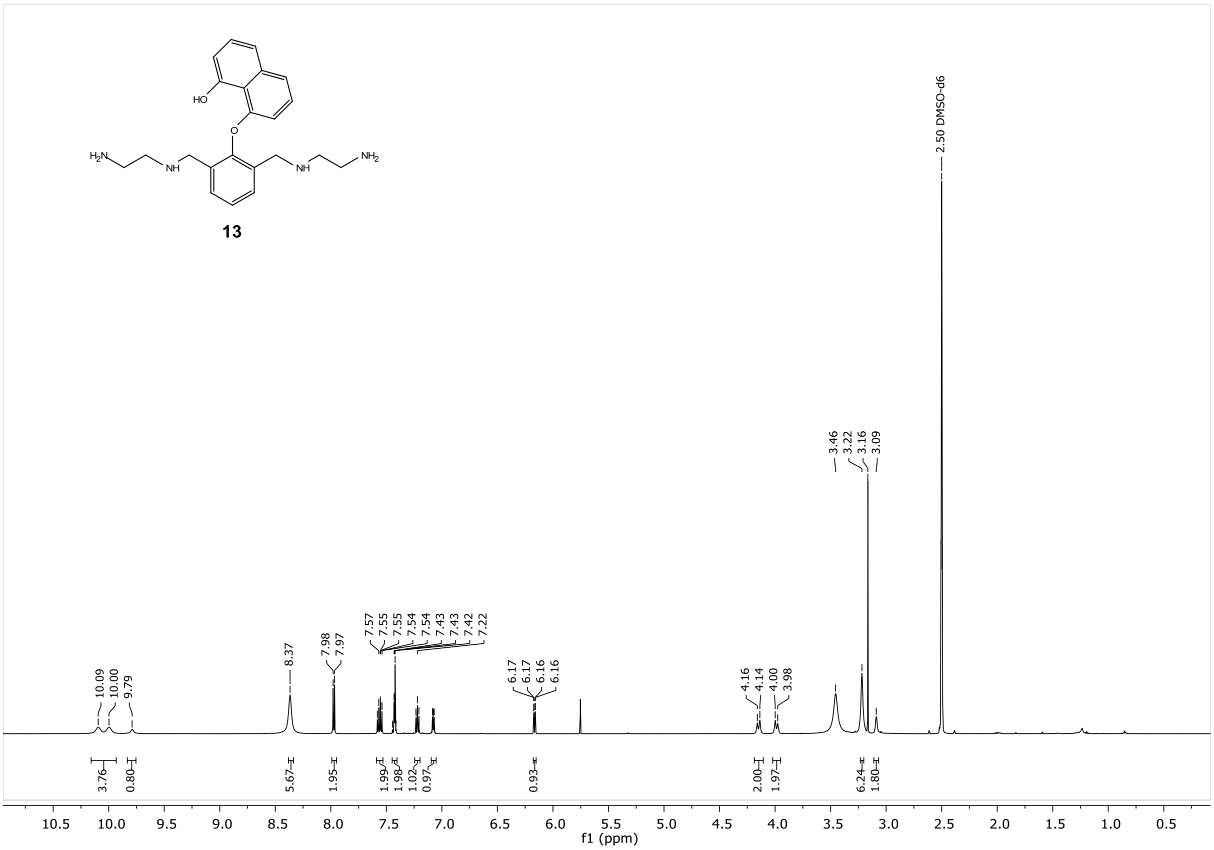

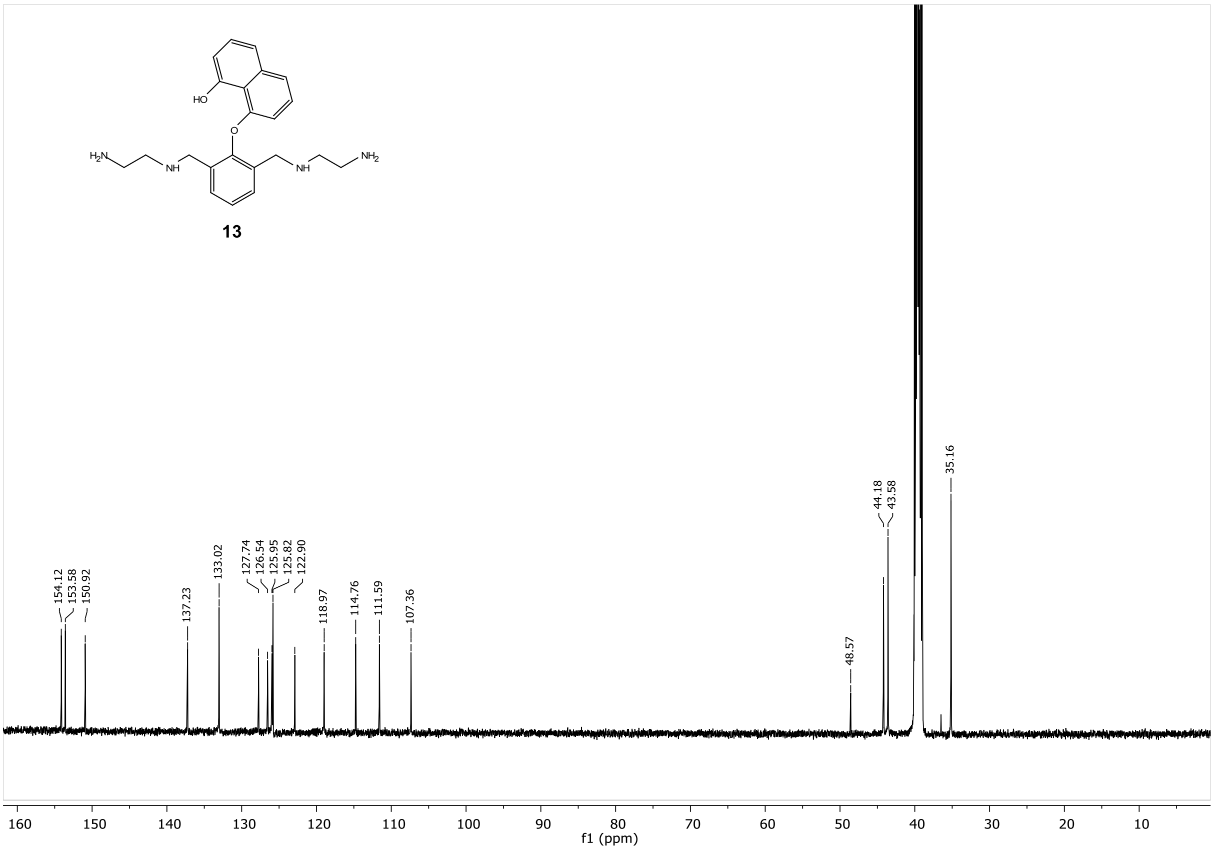

Compound S2

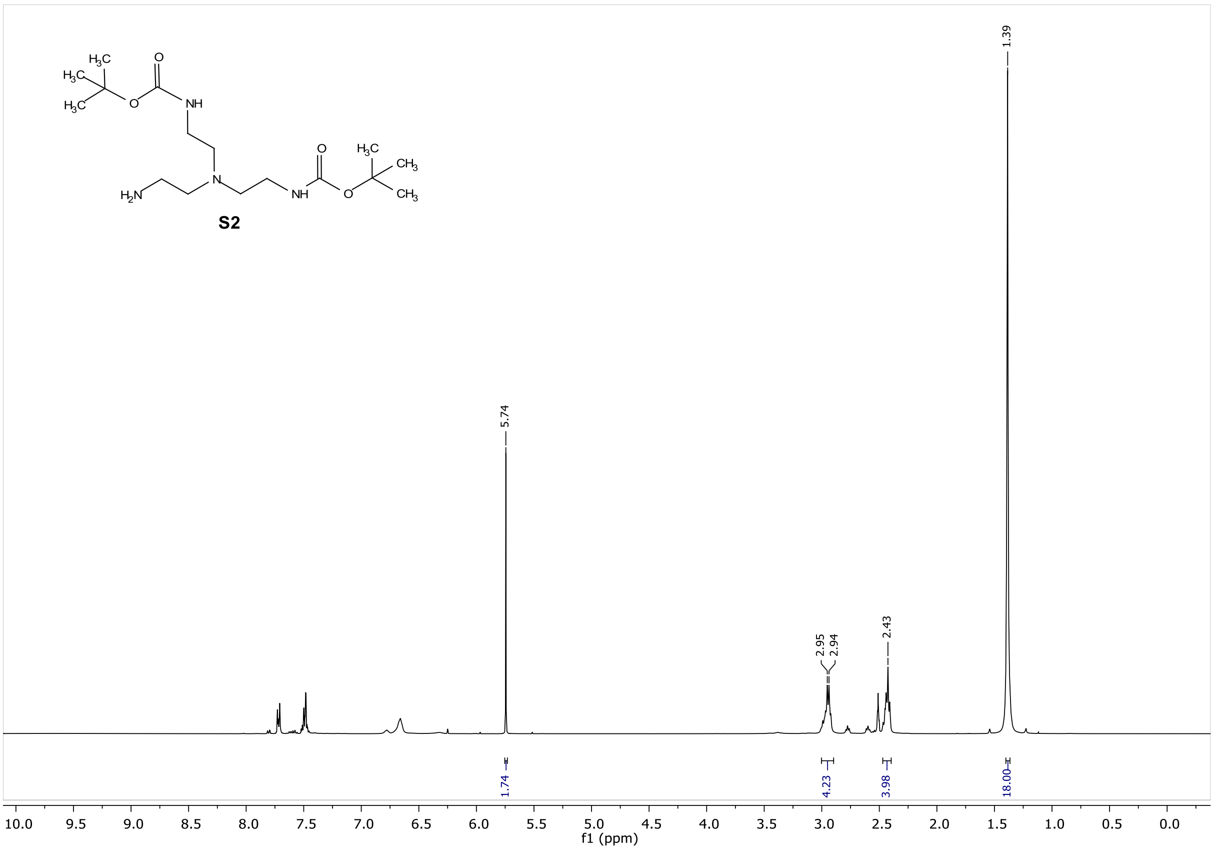

S2
1.39
5.74
2.95
2.94
2.43
1.74
4.23
3.98
18.00
10.0 9.5 9.0 8.5 8.0 7.5 7.0 6.5 6.0 5.5 5.0 4.5 4.0 3.5 3.0 2.5 2.0 1.5 1.0 0.5 0.0
f1 (ppm)

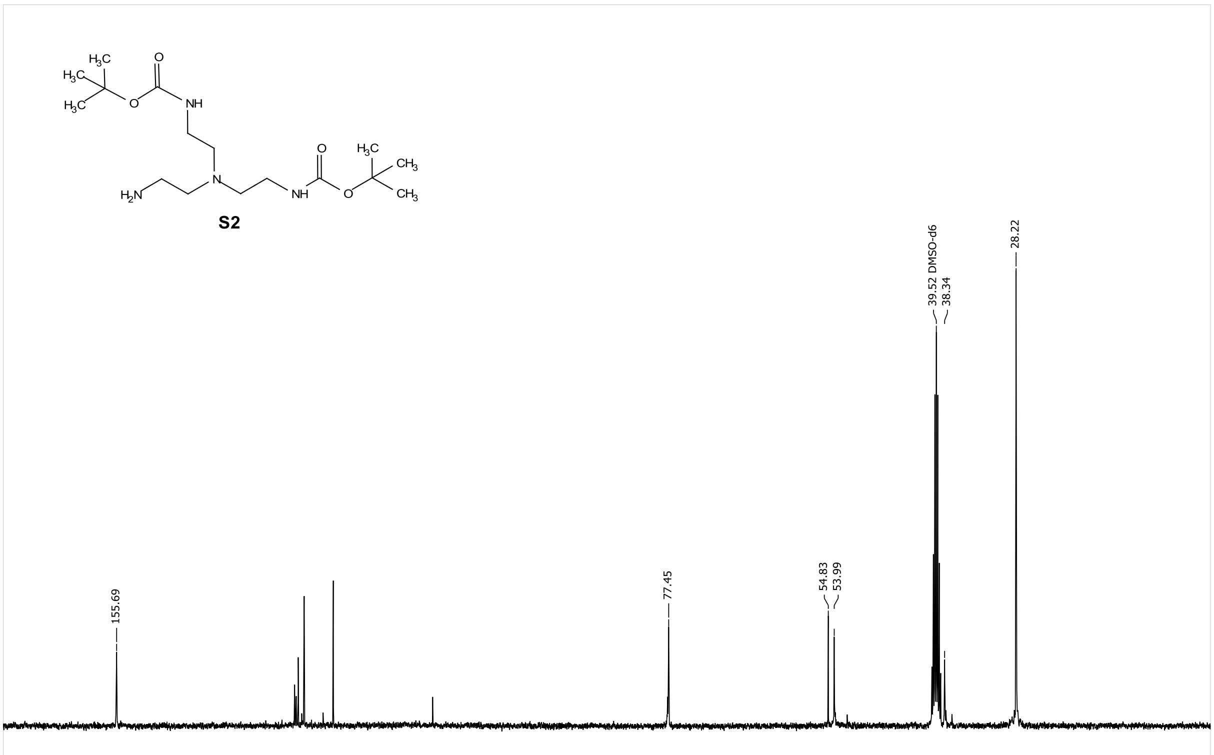

S2
155.69
77.45
54.83
53.99
39.52 DMSO-d6
38.34
28.22
170 160 150 140 130 120 110 100 90 80 70 60 50 40 30 20 10
f1 (ppm)

Compound 14

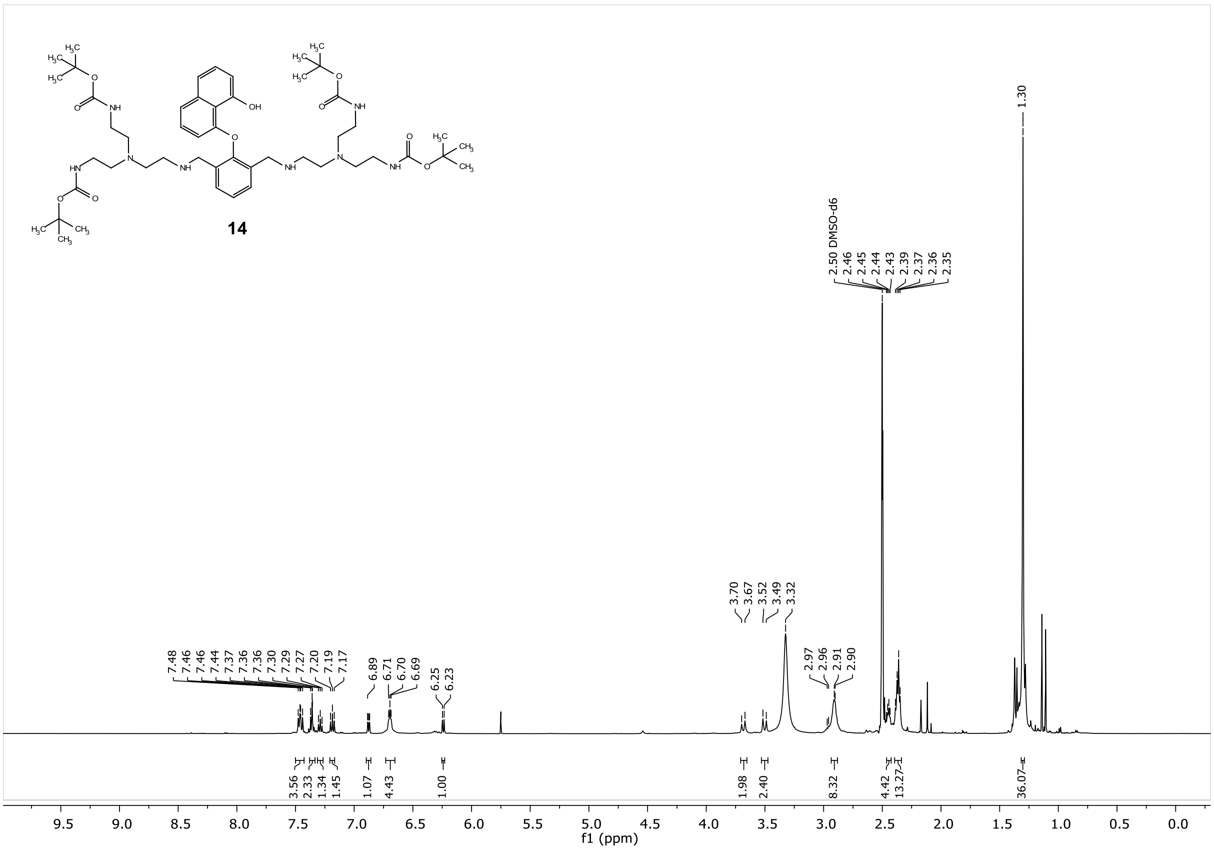
14
1.30
2.50 DMSO-d6
2.46
2.45
2.44
2.43
2.39
2.37
2.36
2.35
3.70
3.67
3.52
3.49
3.32
2.97
2.96
2.91
2.90
7.48
7.46
7.46
7.44
7.37
7.36
7.36
7.30
7.29
7.27
7.20
7.19
7.17
6.89
6.71
6.70
6.69
6.25
6.23
3.56
2.33
1.34
1.45
1.07
4.43
1.00
1.98
2.40
8.32
4.42
13.27
36.07
f1 (ppm)

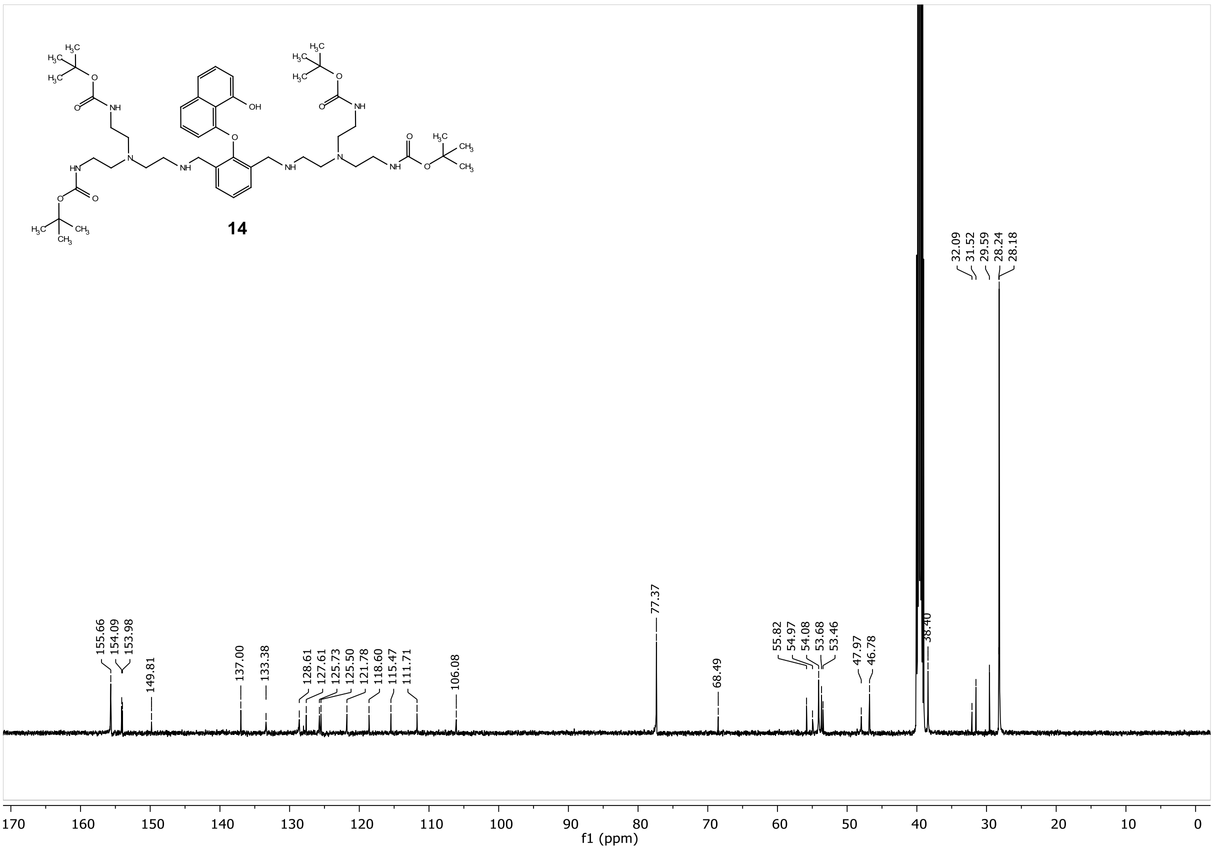
14
32.09
31.52
29.59
28.24
28.18
77.37
155.66
154.09
153.98
149.81
137.00
133.38
128.61
127.61
125.73
125.50
121.78
118.60
115.47
111.71
106.08
68.49
55.82
54.97
54.08
53.68
53.46
47.97
46.78
38.40
f1 (ppm)

Compound 15

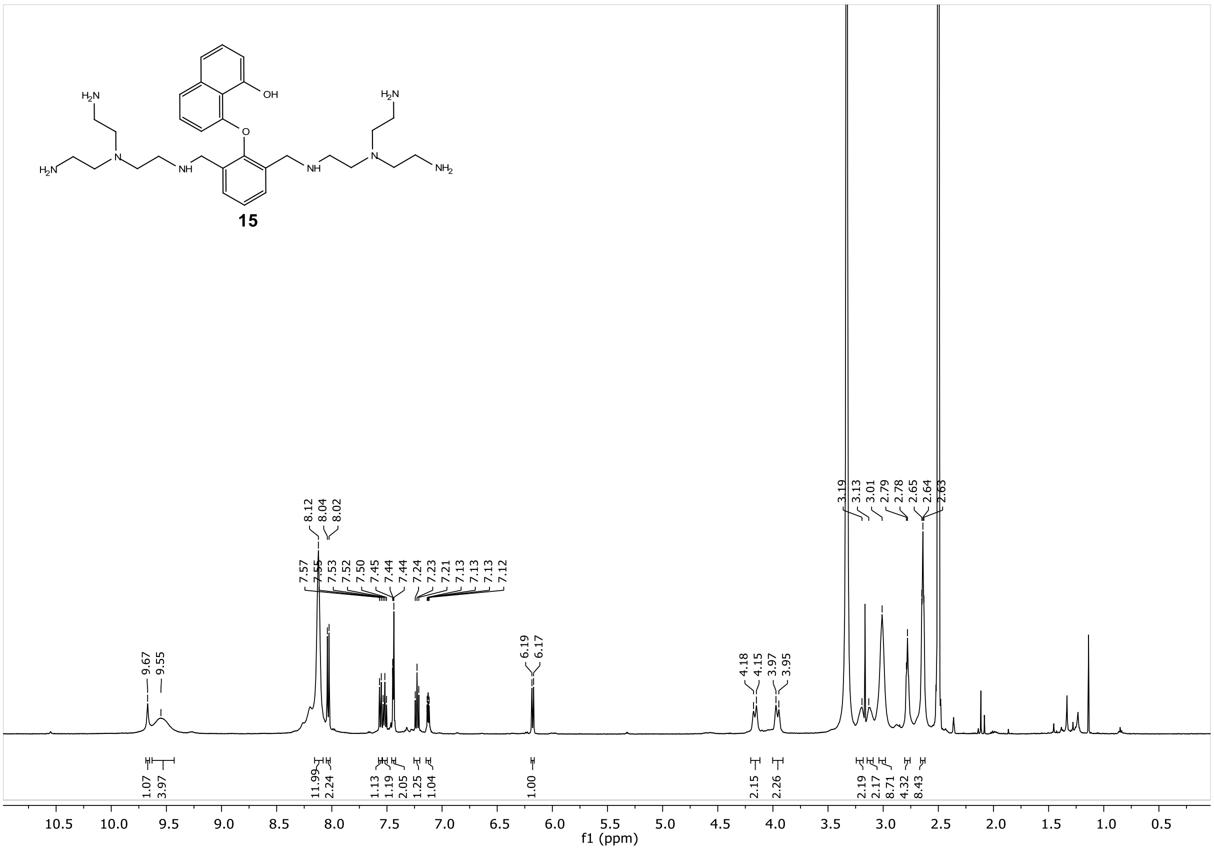

OH
O
H2N
NH
NH
NH2
N
15
9.67
9.55
8.12
8.04
8.02
7.57
7.55
7.53
7.52
7.50
7.45
7.44
7.44
7.24
7.23
7.21
7.13
7.13
7.13
7.12
6.19
6.17
4.18
4.15
3.97
3.95
3.19
3.13
3.01
2.79
2.78
2.65
2.64
2.63
1.07
3.97
11.99
2.24
1.13
1.19
2.05
1.25
1.04
1.00
2.15
2.26
2.19
2.17
8.71
4.32
8.43
f1 (ppm)

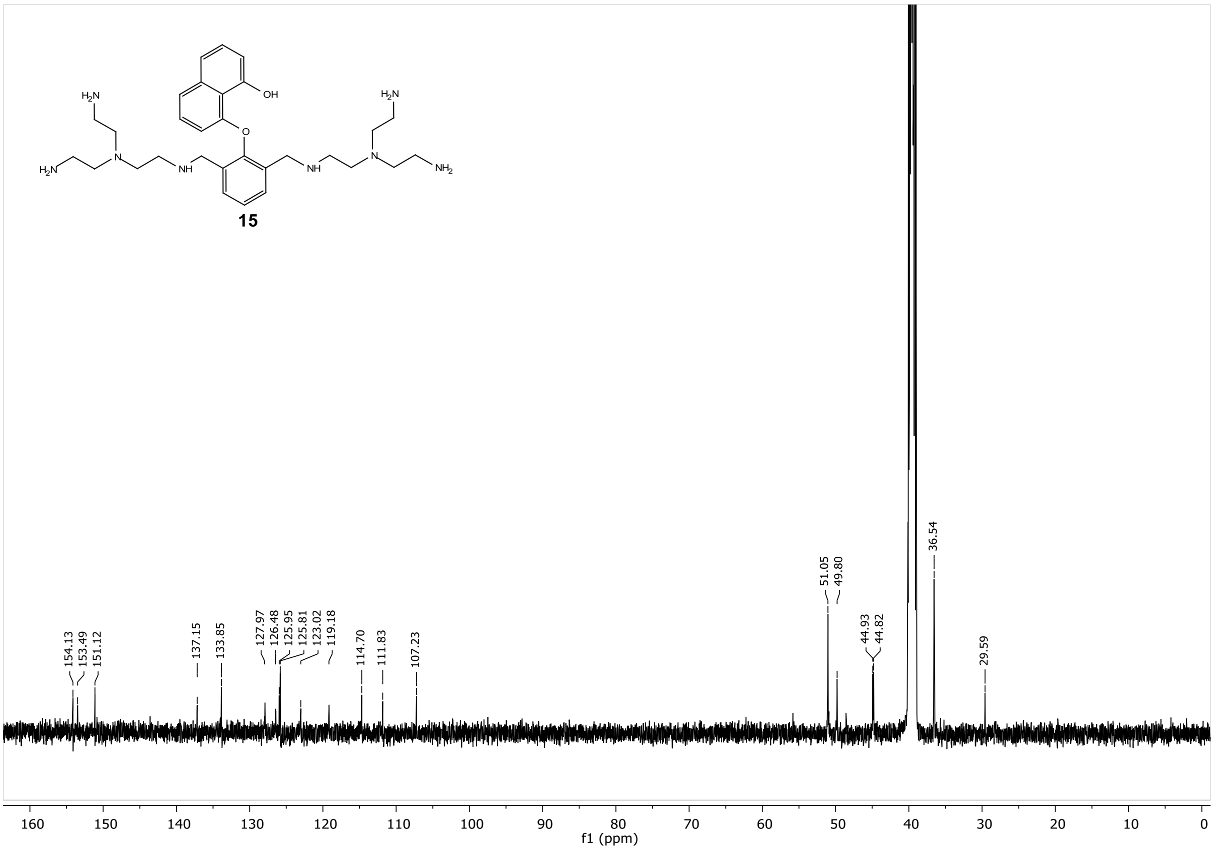

15
154.13
153.49
151.12
137.15
133.85
127.97
126.48
125.95
125.81
123.02
119.18
114.70
111.83
107.23
51.05
49.80
44.93
44.82
36.54
29.59
f1 (ppm)

Compound S3 (Contains traces of NEt_3)

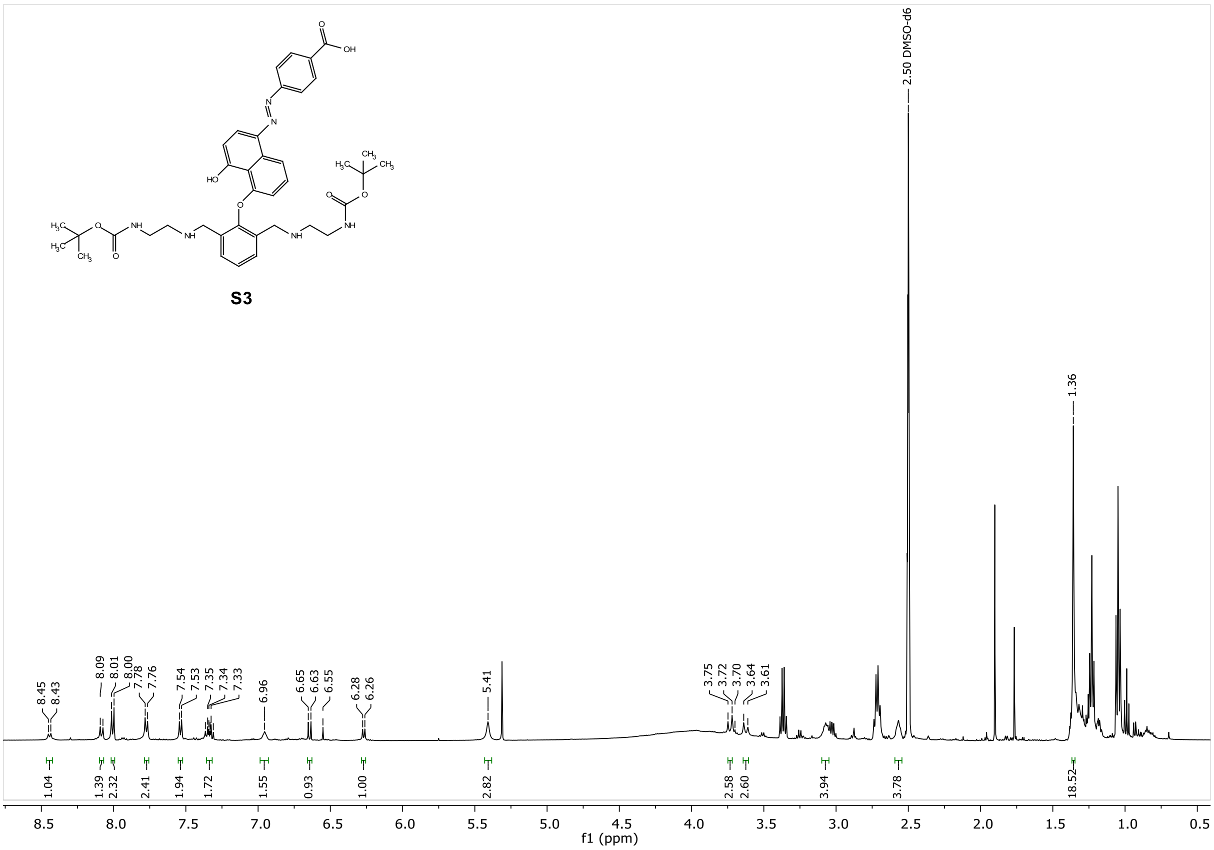

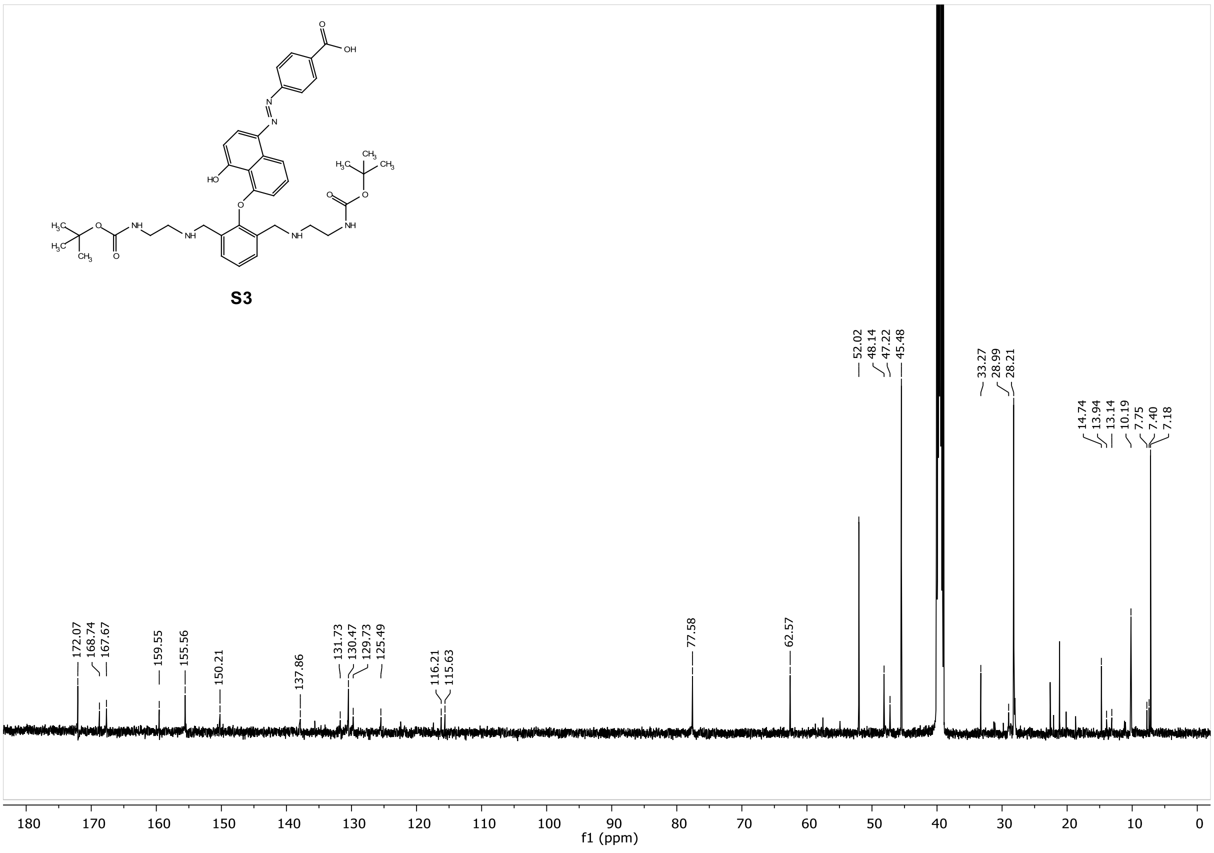

Compound 16 (in D_2O)

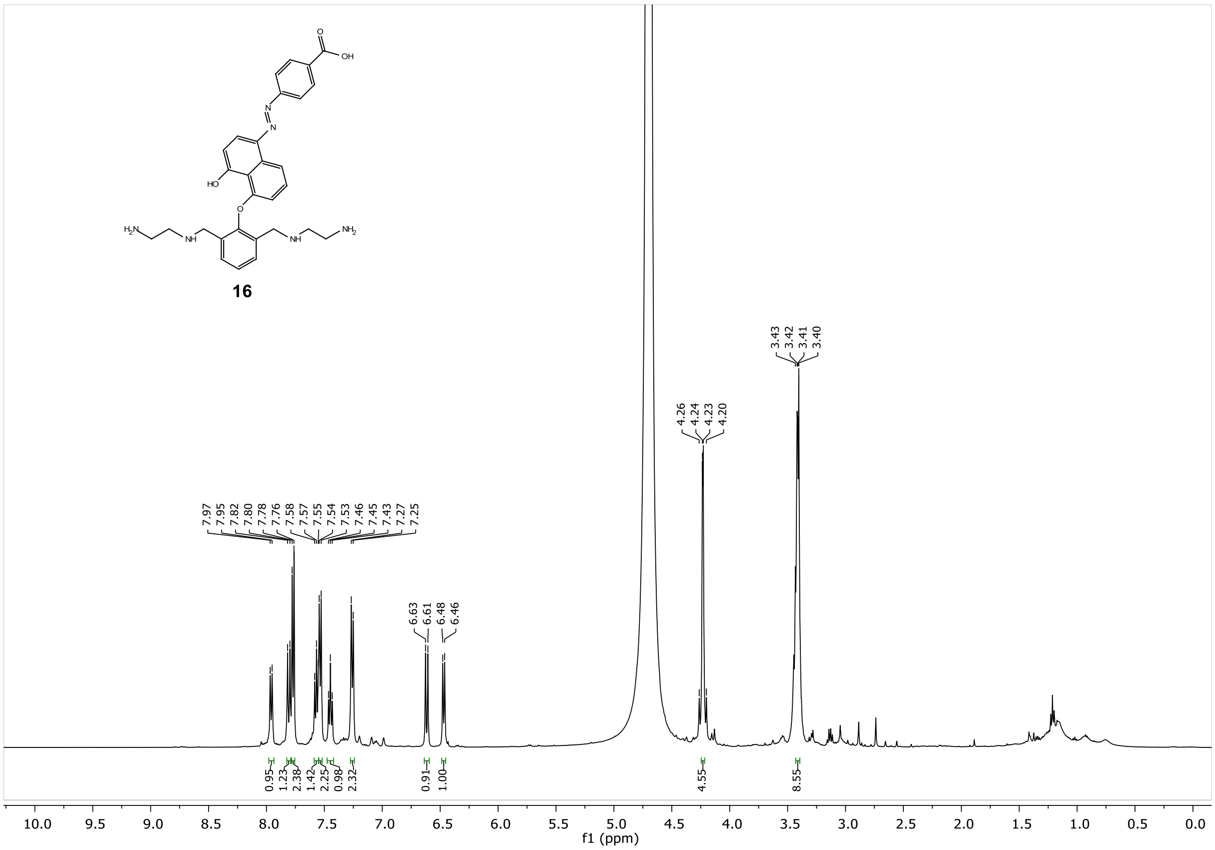

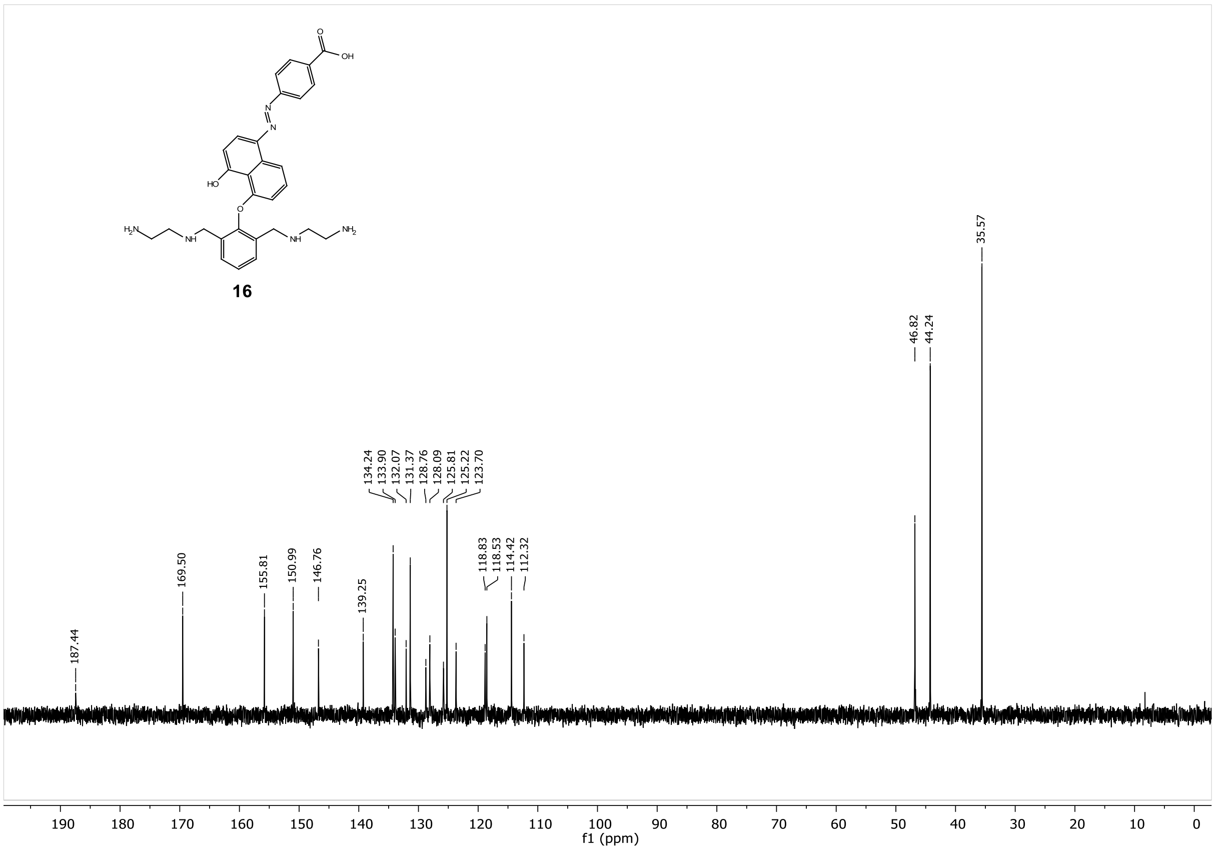

Compound S4

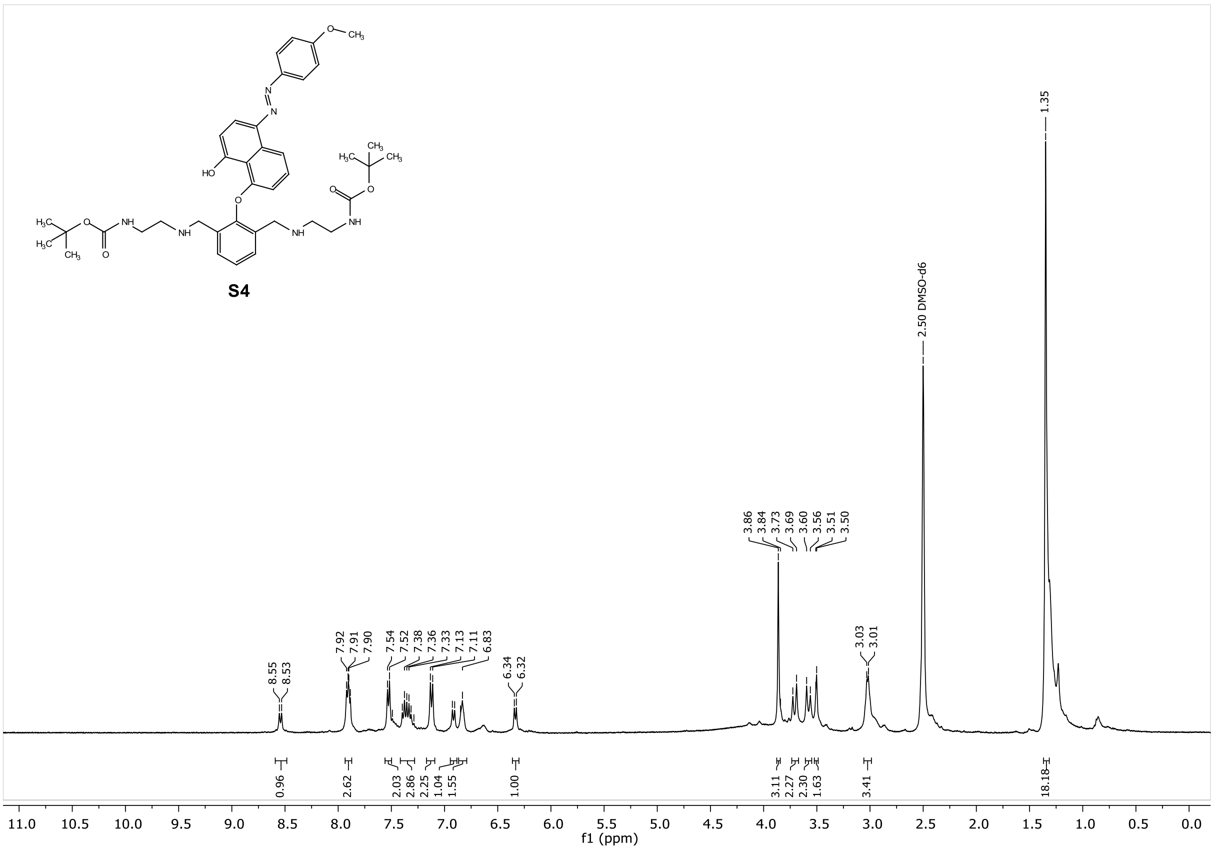
S4
1.35
2.50 DMSO-d6
8.55
8.53
7.92
7.91
7.90
7.54
7.52
7.38
7.36
7.33
7.13
7.11
6.83
6.34
6.32
3.86
3.84
3.73
3.69
3.60
3.56
3.51
3.50
3.03
3.01
0.96
2.62
2.03
2.86
2.25
1.04
1.55
1.00
3.11
2.27
2.30
1.63
3.41
18.18
f1 (ppm)

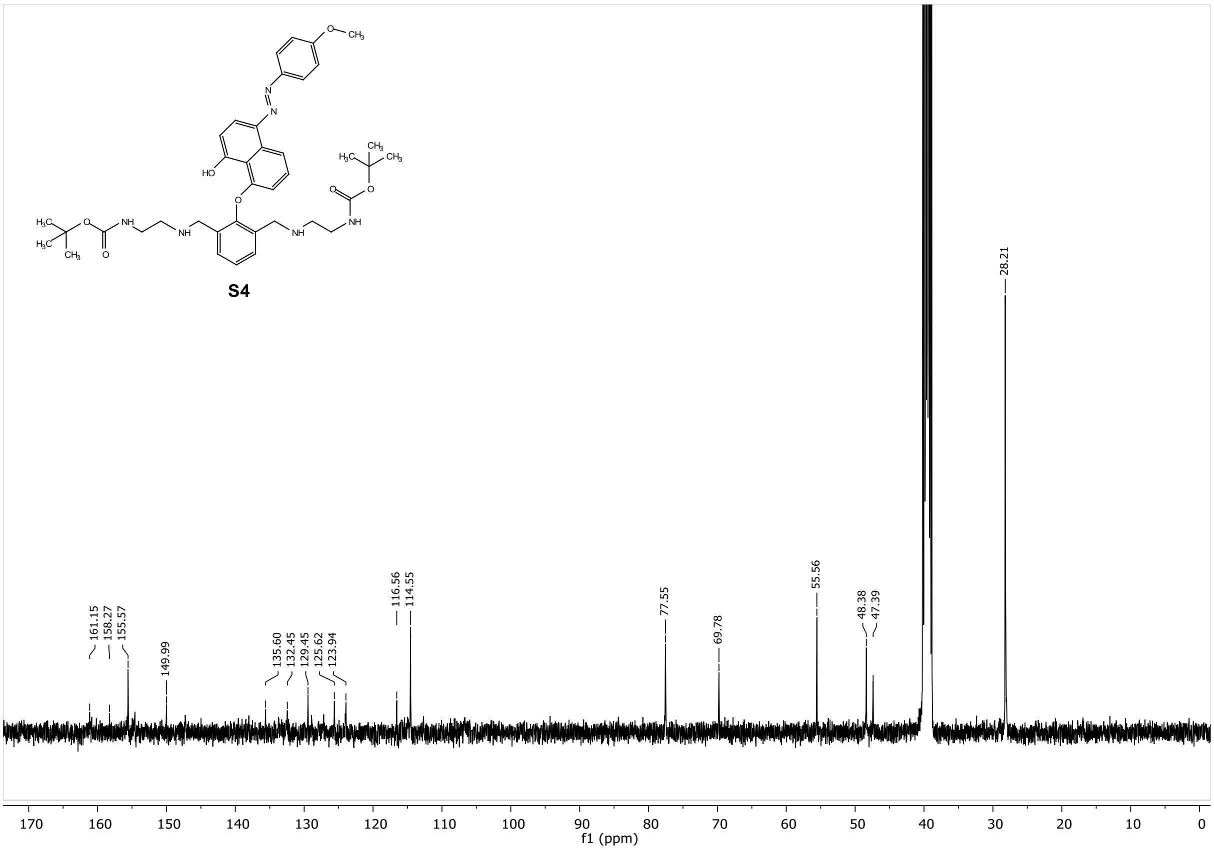
S4
28.21
161.15
158.27
155.57
149.99
135.60
132.45
129.45
125.62
123.94
116.56
114.55
77.55
69.78
55.56
48.38
47.39
f1 (ppm)

Compound 17 (in D_2O)

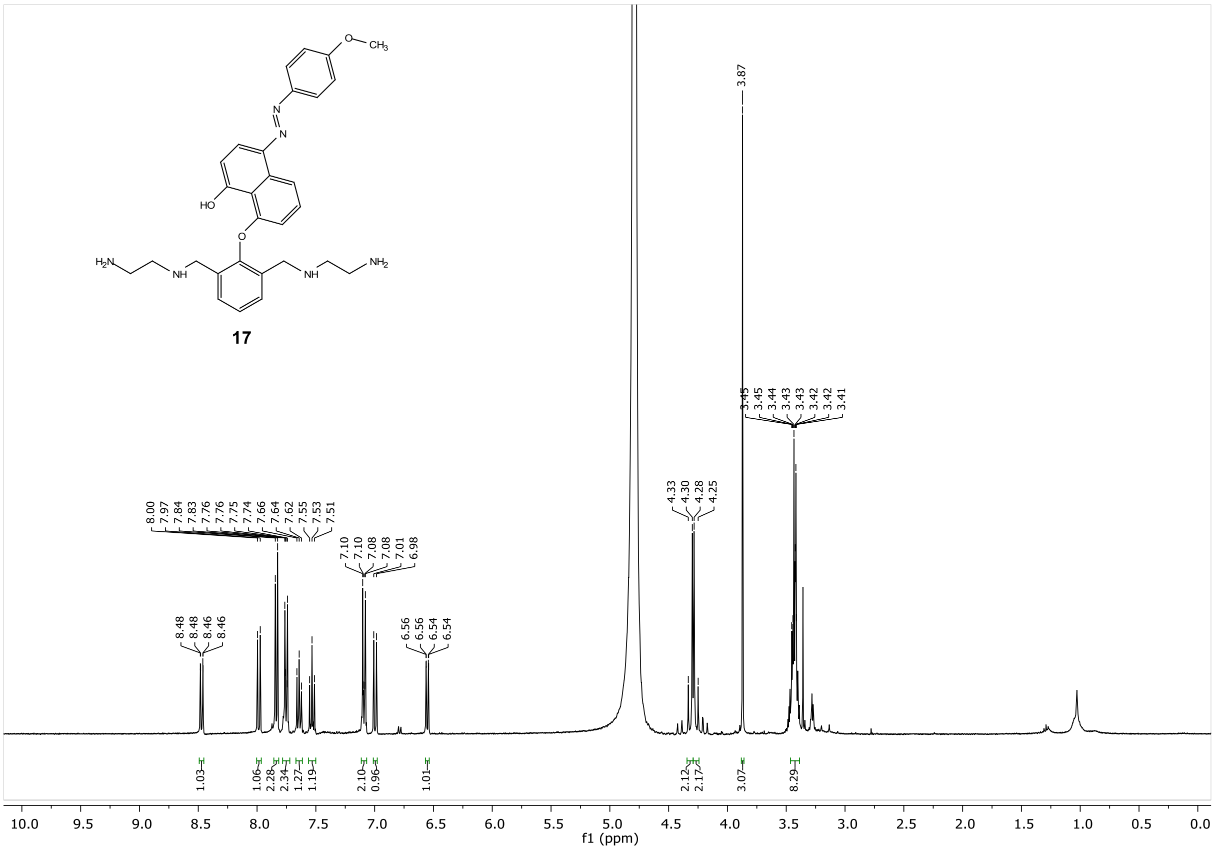

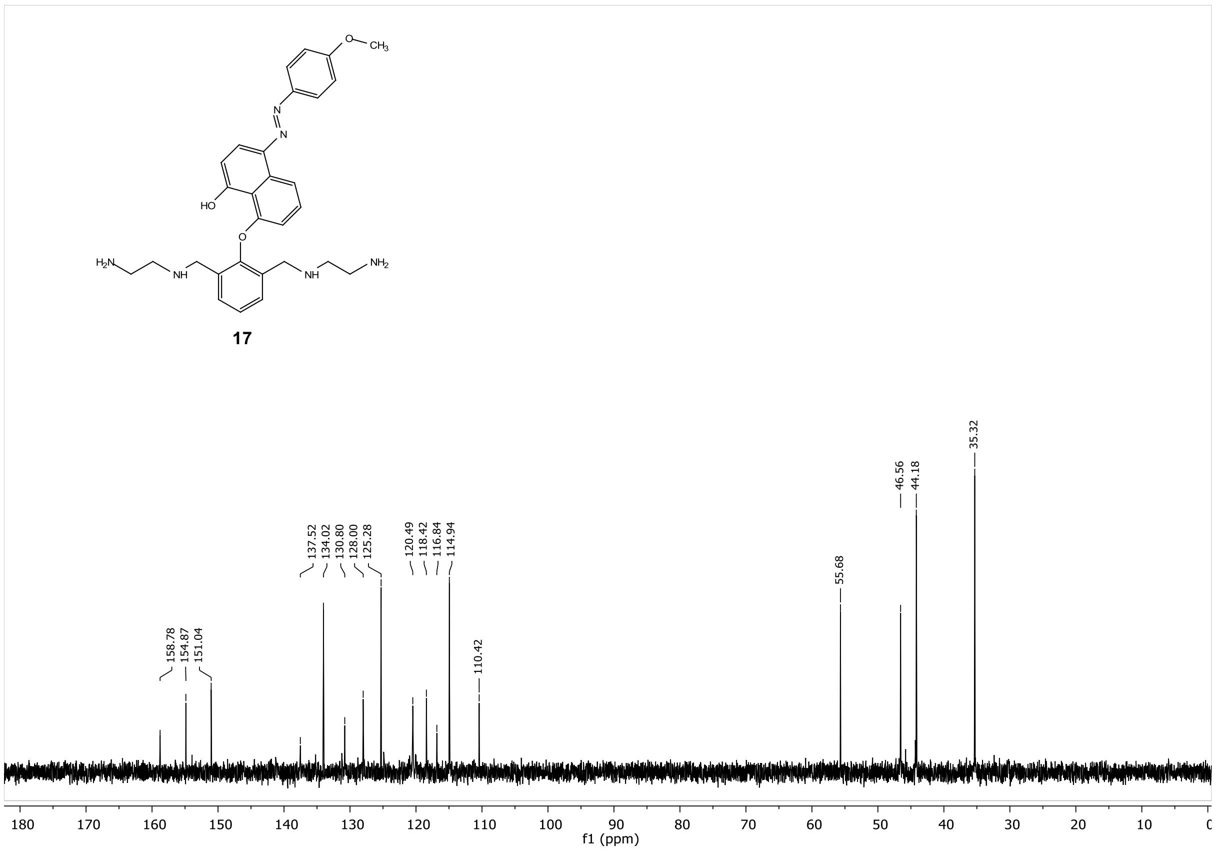

References

[1] M. E. Branum, A. K. Tipton, S. Zhu, L. Que, *J. Am. Chem. Soc.* **2001**, *123*, 1898–1904.

[2] T. D. Krizan, J. C. Martin, *J. Org. Chem.* **1982**, *47*, 2681–2682.

[3] J. P. Ragot, C. Steeneck, M.-L. Alcaraz, R. J. K. Taylor, *J. Chem. Soc., Perkin Trans. 1* **1999**, 1073–1082.

[4] A. G. M. Barrett, F. Blaney, A. D. Campbell, D. Hamprecht, T. Meyer, A. J. P. White, D. Witty, D. J. Williams, *J. Org. Chem.* **2002**, *67*, 2735–2750.

[5] S. Ullrich, Z. Nazir, A. Büsing, U. Scheffer, D. Wirth, J. W. Bats, G. Dürner, M. W. Göbel, *ChemBioChem* **2011**, *12*, 1223–1229.

[6] D. Muller, I. Zeltser, G. Bitan, C. Gilon, *J. Org. Chem.* **1997**, *62*, 411–416.

[7] U. Scheffer, A. Strick, V. Ludwig, S. Peter, E. Kalden, M. W. Göbel, *J. Am. Chem. Soc.* **2005**, *127*, 2211–2217.
